# Instrumented assessment of joint neuromechanics in upper motor neuron syndrome: a systematic review of methods, reliability, validity, and clinical relevance

**DOI:** 10.1186/s12984-026-02108-1

**Published:** 2026-07-29

**Authors:** Karien ter Welle, Alfred C. Schouten, Winfred Mugge, Jonathan C. van Zanten, Erwin E. H. van Wegen, Arno H. A. Stienen, Carel G. M. Meskers, Mark van de Ruit

**Affiliations:** 1 Department of Biomechanical Engineering, Delft University of Technology, Mekelweg 2, Delft, 2628CD The Netherlands; 2 Rehabilitation Medicine, Amsterdam UMC Location Vrije Universiteit Amsterdam, De Boelelaan 1117, Amsterdam, 1081HV The Netherlands; 3 Amsterdam Movement Sciences, Rehabilitation and Development, Amsterdam UMC Location Vrije Universiteit Amsterdam, De Boelelaan 1117, Amsterdam, 1081HV The Netherlands; 4 Amsterdam Neuroscience, Neuroinfection and Inflammation, Amsterdam UMC Location Vrije Universiteit Amsterdam, De Boelelaan 1117, Amsterdam, 1081HV The Netherlands

**Keywords:** Stroke, Upper motor neuron syndrome, Neuromechanics, Instrumented, Quantification, Modeling, Identification, Spasticity, Stiffness

## Abstract

Instrumented neuromechanical assessment (I-NMA) is a promising method for quantifying joint neuromechanics in upper motor neuron syndrome (UMNS), using controlled joint manipulation such as stretch- or continuous perturbations. Compared to traditional clinical assessments, I-NMA can provide improved insight by better separating neural and mechanical contributions and increasing objectivity. However, I-NMA has not yet found its way into clinical practice, possibly due to methodological shortcomings. This systematic review analyzed 74 studies on I-NMA using controlled perturbation in UMNS, examining experimental protocols, input–output analyses to quantify neuromechanical outcome parameters, and their reliability and validity. Most studies employed only single-joint (n=64 studies) assessments, solely under passive conditions (n=63), which overlook clinically relevant aspects such as multi-joint interactions and active control. Among neuromechanical parameters, joint stiffness and reflexive resistance were quantified most, whereas joint dampingand intrinsic neural drive contributions remained underrepresented. Four main input–output analysis methods were recognized: System Quantification (n=55), System Modeling (n=12), and System Identification via impulse (IRF, n=7), or frequency response functions (FRF, n=6). The first quantified passive stiffness during a slow joint stretch and reflexive resistance during a fast stretch. The latter three quantified neuromechanical parameters by fitting models, comprising time-delayed reflex components and intrinsic joint stiffness and damping, either directly using input–output data (System Modeling) or via initial nonparametric IRF or FRF estimation. The reliability of quantified parameters showed substantial room for improvement, especially in the minimal detectable change, ranging from 20% to 947% of the parameter value across analyses. I-NMA outcome measures showed stronger validity when assessed against expected UMNS-healthy differences than when assessed against clinical scales. Future I-NMA development may benefit from improved reliability through protocol consistency and optimized analysis, stronger evidence for the valid separation of neural and mechanical constructs, and enhanced clinical relevance by using active and passive assessments, capturing both paresis and hyperresistance.

**Supplementary Information:**

The online version contains supplementary material available at https://doi.org/10.1186/s12984-026-02108-1.

## Introduction

A lesion to the upper motor neurons in upper motor neuron syndrome (UMNS) is characterized by a loss of neuromuscular control. UMNS can arise from various neurological disorders, such as stroke, cerebral palsy, and multiple sclerosis, of which stroke is the most prevalent cause (160 million disability-adjusted life years globally in 2021 [1, 2]). The loss of neuromuscular control in UMNS results from both neural changes and mechanical changes in muscles and surrounding tissues (BOX 1) [1, 3]. The neural changes involve a reduction in voluntary muscle control, causing paresis, and an increase in involuntary muscle activation, causing hyperresistance^1^ [1]. Mechanical changes, such as muscle atrophy, muscle shortening, and increased mechanical tissue stiffness, further contribute to the effects of paresis and hyperresistance [1, 4]. The combined and intertwined neural and mechanical changes translated to the level of human joints [4] can be encapsulated under the umbrella term ’joint neuromechanics’.

Joint neuromechanics describe the two-way mechanical interaction at a joint, in which an externally applied torque drives joint rotation, and an imposed rotation causes a resistance torque. Intact neuromuscular control enables humans to modulate joint neuromechanics in a task-dependent manner, as required to permit movement or maintain specific postures. In UMNS, the coexistence of paresis and hyperresistance hinders voluntary control of joint neuromechanics, causing difficulty in limb movement [5], abnormal joint postures [1], and limitations in (self-)care and task execution.

For optimal, personalized, and effective UMNS treatment, an in-depth quantification of changes in joint neuromechanics is required, beyond the general information provided by current clinical assessment [6–8]. On the level of joint functions and structures, clinical scales such as the Motricity Index [9] or Manual Muscle Testing [10] are used to evaluate paresis, and the ’Modified Ashworth Scale’ (MAS) [11] or ’Modified Tardieu Scale (MTS)’ [12] to evaluate hyperresistance. These types of clinical assessments are criticized for their heterogeneity in definitions, ordinal scoring, varying objectivity and reliability, and showing floor or ceiling effects [10, 12–14]. Furthermore, these assessments are unable to distinguish between the contributions of the various neural and mechanical constructs (BOX 1), but rather provide some ’lumped’ measure of joint resistance or performance [13, 15–18]. This lumped assessment hinders the prescription and evaluation of treatment specifically targeted at neural [19] or mechanical [20] constructs.

Instrumented neuromechanical assessment (I-NMA) of joints in UMNS could provide a solution to these shortcomings [17, 21, 22]. I-NMA techniques use devices to apply controlled rotations or torques, often referred to as ’perturbations’, to a joint and measure the response. Subsequently, the relationship between the imposed input (e.g., joint rotations or torques) and measured outputs (e.g., joint rotations, torques, or electromyography (EMG)) can be used to quantify joint neuromechanical parameters. I-NMA promotes consistency and objectivity, thereby enhancing the reliability of assessment [23]. Furthermore, by using specific analyses to separate different neural and mechanical joint properties, I-NMA can provide improved diagnostic value [24, 25].

I-NMA has yet to find its way into clinical practice, despite recognition of its advantages in terms of improved construct separation and objectivity [17, 22]. Previous reviews on I-NMA have highlighted key considerations regarding the optimal experimental protocol (e.g., the robotic control scheme and assessment protocol) [17], the most appropriate analyses for separating neural and mechanical constructs [25], and the lack of evidence for reliable and valid neuromechanical parameter quantification [24, 26]. However, aforementioned reviews mostly address one of these aspects of I-NMA, while in practice, these aspects are interconnected. Furthermore, research focuses mostly on I-NMA of hyperresistance (’spasticity’) under passive conditions, while disregarding the paresis component of UMNS. This systematic review, therefore, aims to provide a comprehensive overview of I-NMA in UNMS, identifying: (1) the experimental protocol used for I-NMA using controlled perturbations, (2) the different input–output analyses for quantifying neuromechanical parameters, and their ability to separate neural and mechanical constructs in the context of both paresis and hyperresistance, and (3) the reported evidence for the reliability and validity of the quantified neuromechanical parameters.

Box 1: Changes of joint neuromechanics in UMNS

**Fig. 1:**
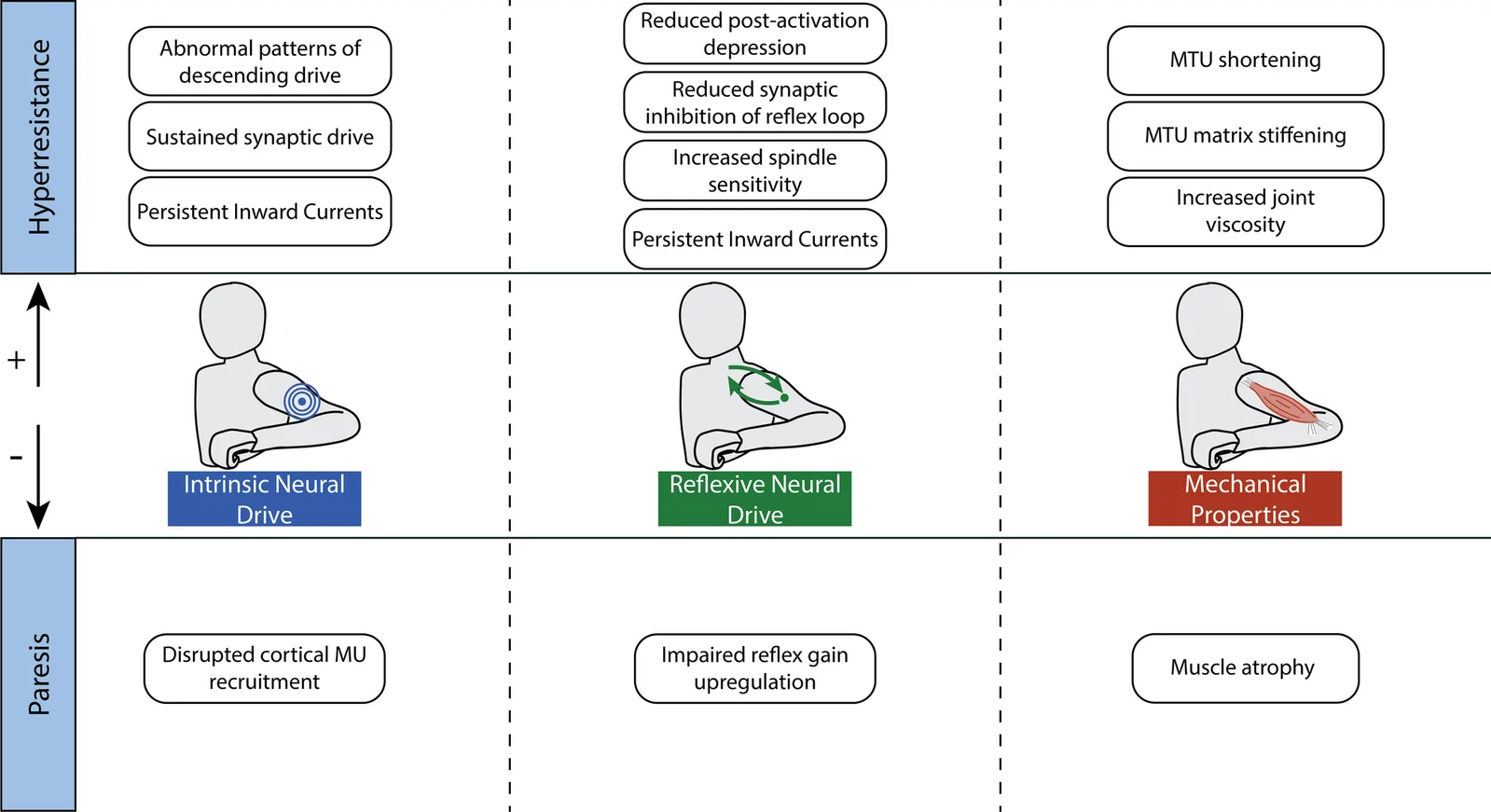
Pathophysiological mechanisms causing changes in joint neuromechanics in Upper Motor Neuron Syndrome (UMNS). The upper panel contains mechanisms causing hyperresistance, and the bottom panel contains mechanisms causing paresis. The three types of contributions to altered joint neuromechanics in UMNS (intrinsic neural drive, reflexive neural drive, and mechanical properties) are represented in the three columns. MTU = muscle tendon unit, MU = motor unit

The separation and quantification of contributions to joint neuromechanics require consistent definitions of pathophysiological mechanisms (Fig. 1), which serve as a clinical framework for the literature reviewed. The separation into paresis and hyperresistance reflects a limited ability to generate resistance during active engagement and excessive resistance present at rest, respectively. Joint neuromechanics are subdivided into three contributions: intrinsic neural drive, reflexive neural drive, and mechanical properties. Intrinsic drive describes a tonic or sustained activation, whereas reflexive drive is characterized as a phasic response to an external perturbation.

**On the level of intrinsic neural drive**, paresis can be caused by a reduced recruitment of motor units due to interrupted pathways [1, 4]. Hyperresistance at rest can be caused by excessive sustained synaptic drive, originating from descending or regional inputs [27, 28]. It may also arise due to an abnormal pattern of descending drive, causing misdirected muscle activation in one muscle group when other muscle groups are engaged; reflecting pathological synergies or co-contraction [27, 29–31]. Furthermore, UMNS is associated with regeneratively activated persistent inward currents (PICs) in spinal motorneurons, which can produce self-sustained plateau potentials outlasting the depolarizing input that triggered them [27, 28, 32]. The combined intrinsic neural drive can give rise to the often-observed UMNS postures, with constantly activated and relatively strong anti-gravity muscles causing a fixed joint position [27].

**On the level of reflexive neural drive**, hyperreflexia contributes to hyperresistance. Hyperreflexia of the Ia-afferent reflex loop is caused by reduced synaptic inhibition as well as reduced post-activation depression and muscle spindle hypersensitivity [4, 27]. Furthermore, the PICs described above can also be triggered by the afferent input generated during a reflex response, resulting in an enlarged and sustained reflexive contribution [33]. Regarding paresis, UMNS patients also exhibit an inability to modulate reflex responses when desired [34, 35].

**On level of mechanical properties**, stiffening of the extracellular matrix [36] and muscle tendon unit (MTU) shortening due to a reduction of sarcomeres in series [3] increase intrinsic resistance. Increase of joint viscosity (e.g., through formation of fluid edemas) adds resistance through damping [37]. Conversely, muscle atrophy due to muscle disuse further contributes to decreased resistance in paresis [38].

Although depicted as three distinct contributions, some cross-influence appears to exist. For example, heightened intrinsic neural drive can bring motor units closer to firing thresholds [34, 35] and MTU shortening can increase spindle sensitivity [4], both of which can heighten reflexive responsiveness.

## Methods

The identification, screening, and data extraction of records were performed according to the PRISMA guidelines for systematic reviews [39].

### Systematic search

The search string consisted of synonyms divided into five categories, extracted from the research aim: ’human joint’, ’neuromechanics’, ’quantification’, ’controlled perturbation’, and ’UMNS’. The synonyms were combined using the OR operator, and the categories were combined using the AND operator. The full search string is shown shown Supplementary Material 1. The search was conducted on June 25th, 2025, in two scientific databases: Web of Science and SCOPUS. Additionally, records were sought through cross-referencing, using the reference lists of identified reports and systematic reviews on similar or related topics

### Article screening and inclusion

Automatic selection tools of the respective databases were used to remove review articles, letters, and book chapters from the set of identified records. An online reference manager (Covidence) was used to automatically remove duplicates.

Articles were considered for review if they (1) described a method of quantifying human joint neuromechanics in UMNS, (2) explicitly aimed to separate neural and mechanical level contributions, and (3) made use of a rotation- or torque-controlled perturbation (e.g., discrete stretches or continuous inputs). Articles published over 20 years prior (before 2005) and articles with small study populations (*<10* participants) were excluded.

Title and abstract screening was performed independently by two authors (KtW and MvdR), with discussion to resolve any disagreements. Records screened as eligible were further assessed based on the full text, where discussion between the same two authors was used to determine the final eligibility.

### Data extraction and synthesis

The results were organized into four subsections: (1) Experimental protocol, (2) Input–output analysis - neuromechanical parameters, (3) Input–output analysis - parameter value extraction, and (4) Reliability & validity. Data for each category were extracted from included studies by a single reviewer (KtW) and tabulated.

The ’experimental protocol’ subsection summarizes the assessed joint, joint angular ranges and the human task instructions. In the ’neuromechanical parameters’ subsection, all reported parameters used to characterize joint neuromechanics were mapped onto a standardized set to facilitate comparison. The ’parameter value extraction’ subsection groups studies by analysis method, with analysis categories defined based on the reviewed studies, and each record assigned to one of these categories. Finally, the ’reliability & validity’ subsection summarizes the reported reliability and validity of studied I-NMAs. The measure with the highest reported reliability or validity was selected when multiple quantifications of the same parameter or construct were available within a study. Both relative and absolute reliability metrics were reported, and multiple validity assessments were reviewed.

## Results

### Selection of studies

**Fig. 2:**
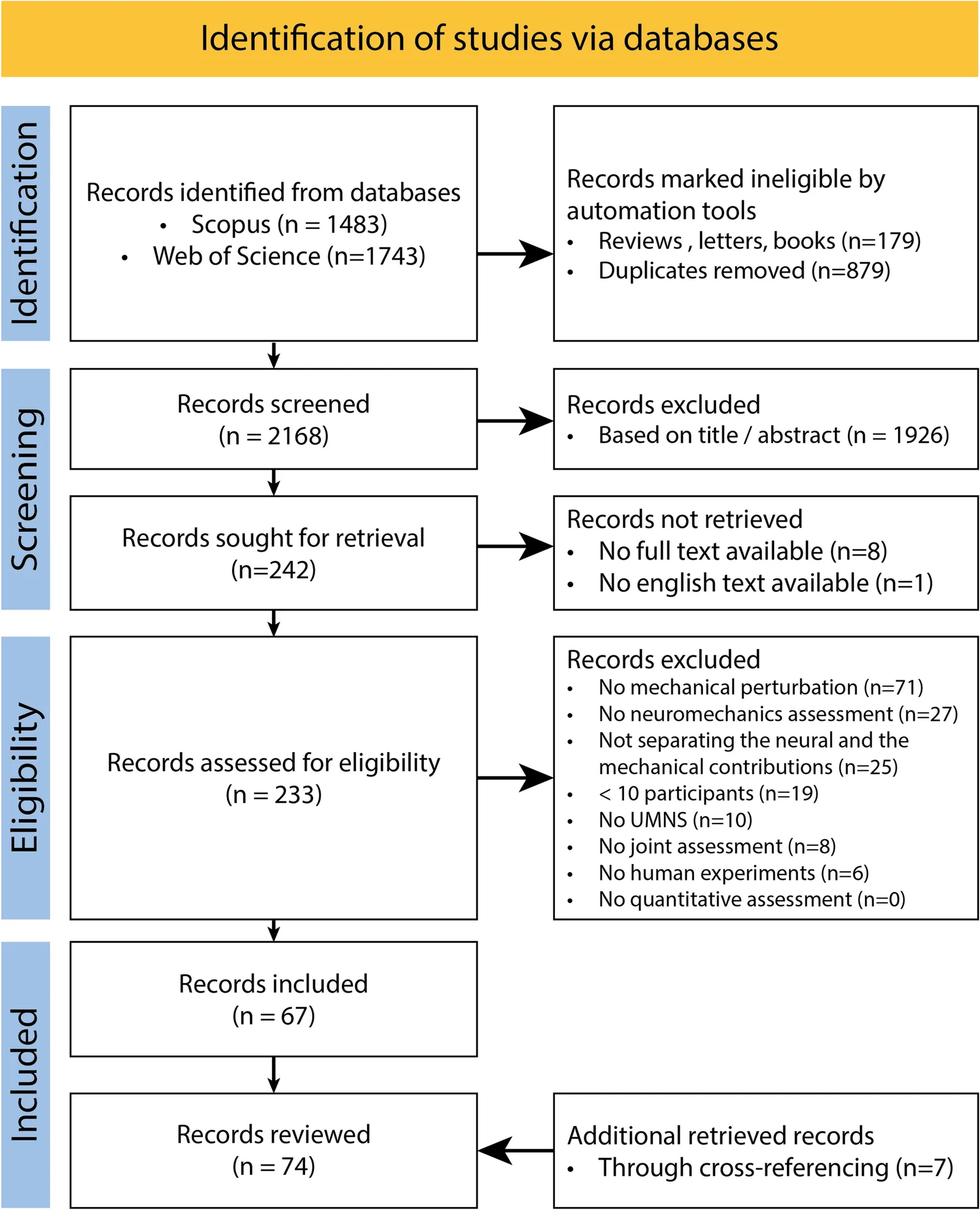
PRISMA flowchart [39], showing the full identification, screening, and eligibility assessment procedure and results

The literature search resulted in 2168 studies after removing review articles, letters, book chapters, and duplicates. Subsequent screening and eligibility assessment left 67 studies (Fig. 2). The addition of seven studies through cross-referencing increased the total to 74 studies included for review [40–113]. All included studies and extracted data are tabulated in Supplementary Material 2.

### Experimental protocol

**Table 1:** A summary of studied neurological disorders and assessed joints

		CVA (C, (S)A, NS)$^{a}$	SCI (C, (S)A, NS)$^{a}$	TBI (C, (S)A, NS)$^{a}$	MS	CP	ALS
	Total$^b$	**50 (33,7,10)**	**14 (12,0,2)**	**3(1,0,2)**	**5**	**11**	**1**
Ankle	**28**	14 (7,1,6)	11 (10,0,1)	3 (1,0,2)	4	3	0
Knee	**13**	3 (3,0,0)	6 (5,0,1)	0	1	3	0
Hip	**5**	1 (1,0,0)	4 (4,0,0)	0	0	0	0
Shoulder	**1**	1 (1,0,0)	0	0	0	0	0
Elbow	**14**	14 (11,0,3)	0	0	0	2	0
Wrist	**24**	21 (12,6,3)	0	0	0	3	1
MCP	**2**	2 (2,0,0)	0	0	0	0	0

Refer to Supplementary Material 2 for individual study details

CVA = Cerebrovascular accident, SCI = Spinal cord injury, TBI = Traumatic brain injury, MS = Multiple sclerosis, CP = Cerebral palsy, ALS = Amyotrophic lateral sclerosis, MCP = metacarpophalangeal.

$^{a}$ Brackets indicate phase since the event: C = Chronic >6 months, (S)A = (Sub)acute <6 months, and NS = Not specified or both phases

$^b$ Total counts for joints and neurological disorders. Due to studies considering multiple disorders or joints, rows/columns do not add up to these totals

Table 1 summarizes the studied joints and neurological disorders in the included literature. The most assessed joints were the ankle [44, 46, 51, 54, 58, 59, 63, 66, 67, 69, 71, 72, 76, 81, 85, 92, 94, 95, 98–101, 103, 104, 106–109] and wrist [40–43, 50, 52, 53, 60, 61, 64, 65, 70, 73, 75, 80, 82, 86, 90, 91, 96, 97, 111–113], followed by the knee [47, 49, 55, 56, 59, 71, 72, 77–79, 92, 93, 102] and elbow [45, 48, 68, 70, 74, 83, 84, 87–89, 103, 105, 107, 110], with lesser attention for the hip [59, 71, 72, 92, 93], shoulder [83], or metacarpophalangeal (MCP) joints [57, 62].

Regarding neurological disorders, most studies investigated patients with a stroke (cerebrovascular accident, CVA) [40–43, 45, 47, 48, 50, 52, 53, 56, 57, 59, 62–66, 68–70, 73–76, 80–91, 96–99, 101, 103–107, 110–112], followed by spinal cord injury (SCI) [51, 54, 55, 58, 66, 67, 71, 72, 77, 81, 92, 108, 109, 114] and cerebral palsy (CP) [43, 49, 60, 61, 78, 79, 87, 89, 94, 95, 100]. Multiple sclerosis (MS) [46, 54, 66, 67, 102], traumatic brain injury (TBI), [44, 81, 101] and amyotrophic lateral sclerosis (ALS) [113] were studied to a lesser extent. The majority of studies included patients in the chronic (>6 months) phase of their disorder.

**Fig. 3:**
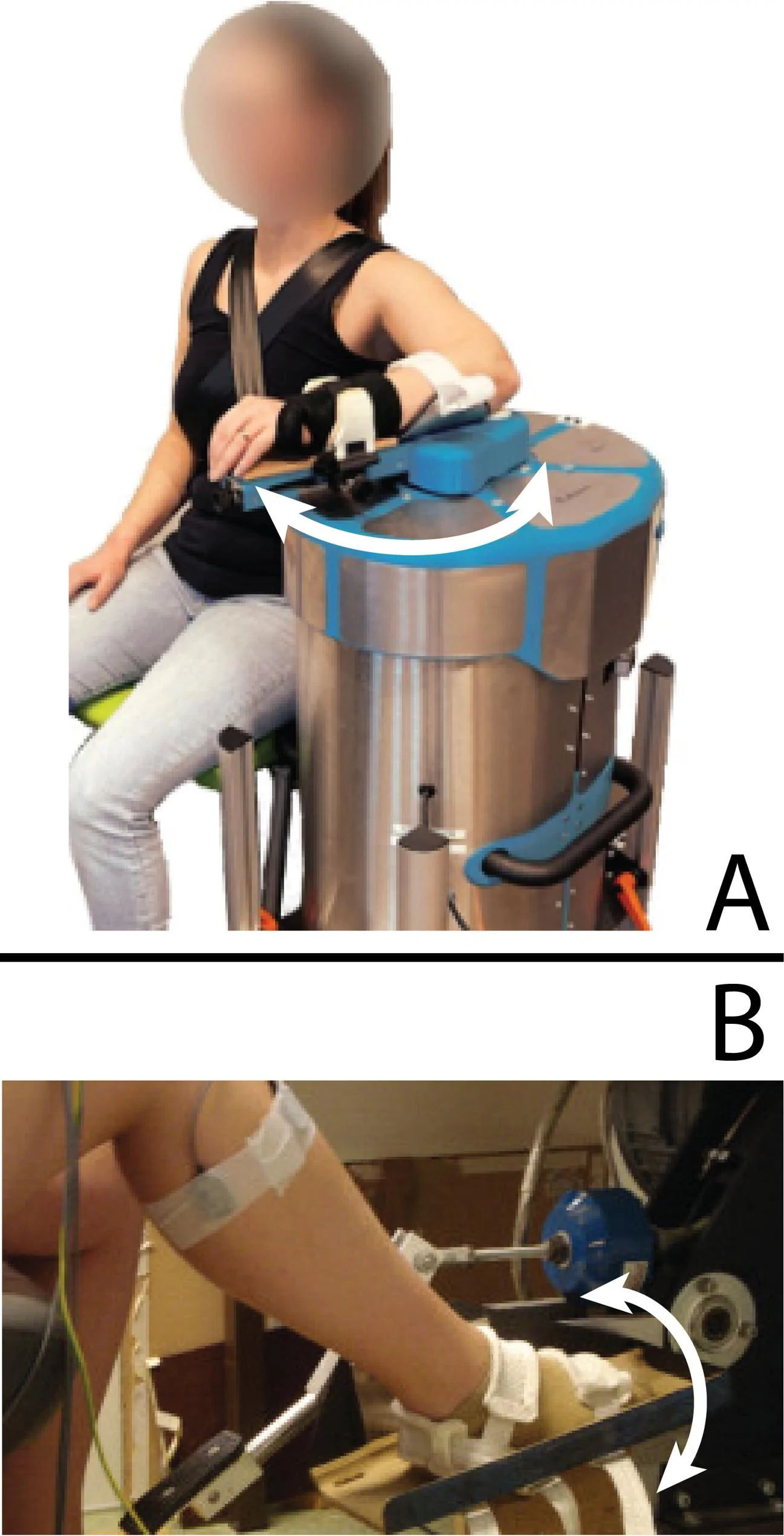
Examples of **A** upper limb [88] and **B** lower limb [95] instrumented neuromechanical assessment. Both pictures are published under the Creative Commons Attribute license (https://creativecommons.org/licenses/by/4.0/)

**Fig. 4:**
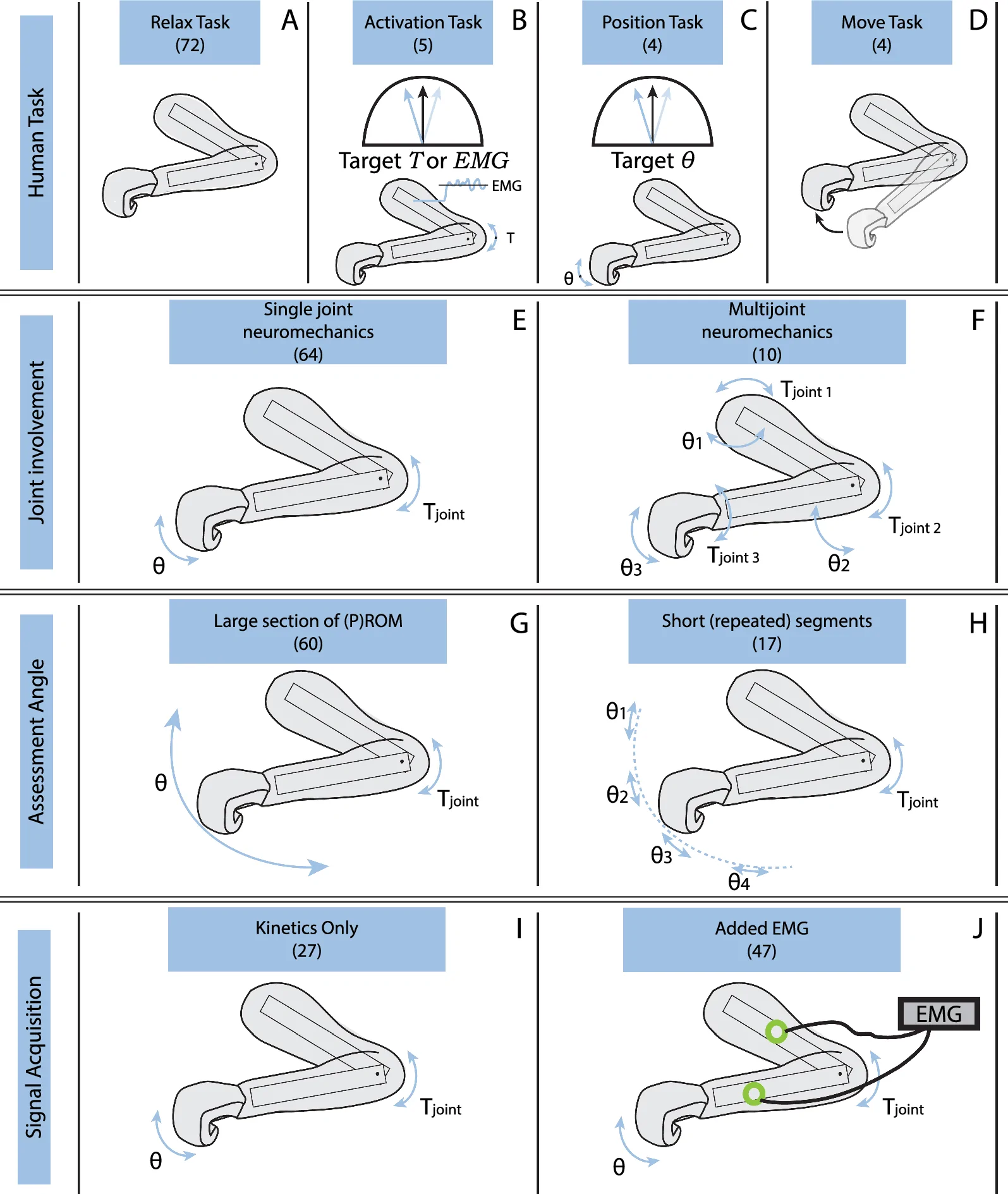
Overview of experimental protocol choices in instrumented neuromechanical assessment across four categories: Human Task (**A**–**D**), Joint Involvement (**E** and **F**), Assessment Angle (**G** and **H**), and Signal Acquisition (**I** and **J**). Number of occurrence across all studies is shown (in brackets). Counts exceed 74 per category as some studies performed multiple analyses. In panels **B** and **C**, gauges illustrate the metric held constant by the participant using visual feedback: torque (*T*), EMG, or joint rotation (*θ *). $$T_{joint} = $$ joint torque, EMG = electromyography, (p)ROM = (passive) Range of Motion. For individual study details, see Supplementary Material 2

All studies used an experimental hardware set-up, with the joint or limb coupled to an instrumented device applying controlled rotational or torque perturbations. Figure 3 shows two customized devices for upper and lower limbs, but the included literature also contained studies using commercially available devices such as the Neuroflexor [40–43, 52, 53, 60, 61, 64, 73, 75, 76, 90, 91], the Biodex dynamometer [50, 82], or the KinCom dynamometer [46]. Principal differences in experimental protocol were identified regarding human task instruction, joint involvement, and assessed joint angle (Fig. 4).

Of the 74 included studies, 72 instructed participants to perform a ’relax’ task as part of their assessment, to assess involuntary hyperresistance (Fig. 4A). For this relax task, 71 studies used an open-loop (OL) human-device interaction [40–98, 100–110, 112]. In this set-up, the mechanical device acts ’infinitely’ stiff and prescribes joint rotation, while the participant aims to give as little resistance as possible (i.e., is relaxed). In 24 of the 72 relax task studies, the relaxed state of the participant was confirmed by the measurement of EMG [46, 54, 55, 57, 58, 64, 66, 68, 74, 77–79, 83, 84, 93–95, 98, 103, 105–109]. Five studies requested the participants to provide a constant joint torque or EMG activation level during device-imposed rotation, in a ’torque’ or ’EMG’ task (Fig. 4B), such as sustained flexor or extensor contraction [86, 101, 102, 110, 113]. An additional four studies used a ’move’ task (Fig. 4D), in which rotational perturbations were applied during (assisted) voluntary movement (e.g., single-joint movements such as flexion or extension, or functional activities such as walking) [59, 72, 83, 99].

Four of the 74 included studies used a ’position’ task, to assess UMNS paresis (Fig. 4C) [86, 87, 111, 113]. Here, participants were requested to actively engage and resist perturbations while maintaining a fixed joint angle. For this task, the device and participant are connected in ’closed-loop’ (CL) and perturbations are applied as torques rather than rotations. The perturbation torque and the human resistance torque, combined, prescribe the joint rotation through the virtual dynamics of the device. The virtual environment in which the resist tasks were executed varied from low impedance to more viscous or damped environments [87, 111, 113, 115].

Among the included studies, 64 focused on the assessment of a single joint at a time (Fig. 4E) [40–56, 58, 60–68, 70, 73–82, 84–86, 88–91, 94–99, 101–113]. A multi-joint approach was adopted in 10 studies (Fig. 4F), either measuring a single joint under the influence of different poses or loading of other joints [57, 69, 87, 100], or simultaneously quantifying neuromechanics of multiple joints during a single assessment [59, 71, 72, 83, 92, 93]. In addition, three of these multi-joint studies also assessed the effect of the contralateral limb posture on joint neuromechanics [59, 71, 93]. The multi-joint and multi-limb assessments demonstrated coupling on the mechanical level in multi-articulate musculature [69, 100], on the level of reflexive neural drive [57, 59, 71, 72, 83, 92, 93], and on the level of intrinsic neural drive through synergistic coupling [57, 87–89].

A total of 60 studies used perturbations spanning almost the full (passive) range of motion ((p)ROM, Fig. 4G) [40–98, 100]. Alternatively, 17 studies used only small amplitude perturbations causing limited joint rotation (*< 15* degrees) [62, 86, 87, 99, 101–113]. Nine studies repeated such a small amplitude experiment across different joint postures (Fig. 4H), to explicitly assess changes over the (p)ROM. All concluded that one or more neuromechanical parameters were sensitive to joint posture [62, 99, 103–109].

Out of the 74 included studies, 27 measured only kinetic signals during assessment (Fig. 4I), while 47 studies added EMG to their data-acquisition protocol (Fig. 4J) [42, 44, 46, 47, 49, 54, 55, 57–59, 62–68, 71, 72, 74, 76, 77, 77, 79, 81, 83, 84, 86, 92, 94–100, 103–111, 113, 114]. EMG was used to confirm correct task execution (in relax or activation tasks) or as data input for the neuromechanical analysis. Among studies using EMG-signal, all but two specified the use of uni- or bipolar surface EMG, and the remaining two [44, 67] did not specify the EMG acquisition method.

### Input–output analysis - neuromechanical parameters

**Fig. 5:**
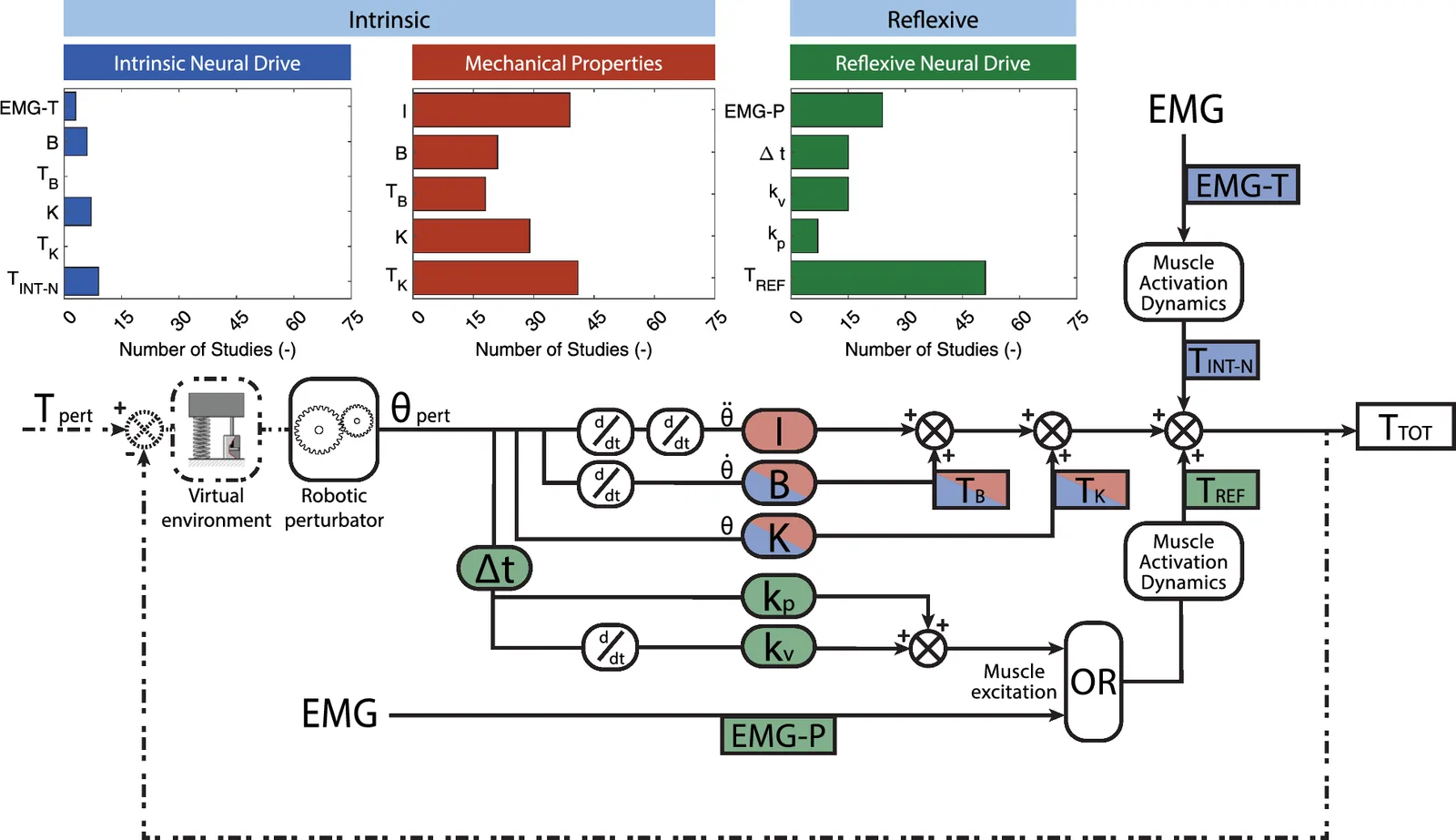
A block diagram of joint neuromechanics, containing parameters used to quantify pathophysiological constructs in upper motor neuron syndrome (UMNS). The color coding of parameters represents a mapping to the UMNS constructs of Fig. 1. The dotted line represents closed loop feedback, which is not present in an open loop set-up. The included bar graph shows the number of occurrences of the parameters in all included studies (74). *θ =* rotation angle, *T =* torque, $T_K =$ stiffness torque, *K =* stiffness, $T_B =$ damping torque, *B =* damping, *I =* inertia, $T_{INT-N} =$ torque from intrinsic neural drive, $T_{REF} =$ reflex torque, $k_p =$ position reflex gain, $k_v =$ velocity reflex gain, *Δ t=* reflex time-delay, $T_{TOT} =$ total resistance torque, *EMG-P* = phasic EMG, *EMG-T* = tonic EMG. $T_K$, $T_B$, *K*, *B* were counted under ’intrinsic neural drive’ if its influence on these parameters was investigated

Using rotation- or torque-controlled perturbation experiments (e.g., stretches or continuous inputs), the included studies identified different sets of neuromechanical parameters, which are summarized into a single model as shown in Fig. 5. Besides the human model, Fig. 5 shows the interaction between the device and the human joint in either an OL (rotation input) or CL (torque perturbations and virtual environment) fashion. The parameters in Fig. 5 adhere to the same grouping as used in BOX 1: intrinsic neural drive, reflexive neural drive, and mechanical properties. While the intrinsic and reflexive neural drive contributors are of neural origin, their effects can also be assessed as mechanical resistance. Accordingly, intrinsic joint resistance reflects both intrinsic neural and mechanical contributions, whereas reflexive joint resistance comprises only reflexive neural contributions. The total measured resistance torque ($T_{TOT}$) is the sum of torques from all intrinsic and reflexive contributions. Note that while the block diagram in Fig. 5 depicts linear multiplication and addition operations for simplicity and interpretability, 25 of the included studies contained some nonlinear operations involving the depicted parameters.

#### Mechanical properties

The parameters used to describe the mechanical part of intrinsic resistance, as a result of passive MTU properties, were an acceleration-responsive inertia $I\, (Nm \cdot s^2/rad)$ [40–43, 52, 53, 59–62, 64, 65, 71–73, 75, 76, 80, 86, 87, 90, 91, 95–99, 101–109, 111–113], velocity-responsive damping $B\, (Nm \cdot s/rad)$ [62, 86, 87, 90, 91, 94, 95, 98, 99, 101–109, 111–113] or damping torque $T_B\, (Nm)$ [40–43, 52, 53, 60–62, 64, 65, 73, 75, 76, 80, 90, 91, 104], and rotation-responsive stiffness *K  (Nm/rad)* [42, 56, 62, 86–91, 94–113] or stiffness torque $T_K\, (Nm)$ [40–43, 45, 46, 48, 50–53, 56–62, 64–69, 71–76, 80–85, 90–93, 104].

#### Reflexive neural drive

In 51 studies, the reflexive contribution was quantified as a reflex torque $T_{REF} (Nm)$; a torque induced by velocity-dependent spinal feedback in response to rapid joint rotation [40–43, 45, 46, 48, 50–53, 56–62, 64–69, 71–76, 80–85, 87–98, 100, 104, 113]. Alternatively, 24 studies parameterized this contribution through quantification of neural reflex excitation: the phasic EMG level of the stretched muscle (*EMG-P, (mV)*). These quantifications ranged from max. amplitude or area under curve of phasic EMG, to counting the occurrence of a pre-specified (e.g., n times standard deviation) change in EMG level from baseline [46, 47, 49, 54, 55, 57–59, 63, 65–67, 71, 72, 74, 76–79, 81, 83, 92, 93, 104]. At a higher detail level, some studies quantified reflex loop feedback parameters, describing contraction as response to joint rotation $k_p\, (Nm/rad\, or\, mV/rad)$ [62, 90, 91, 101, 102, 110, 113] or joint velocity $k_v\, (Nm \cdot s/rad\, or\, mV \cdot s/rad)$ [62, 86, 90, 91, 101–109, 111, 113]. These feedback responses were modeled explicitly using a time delay $\Delta t\, (sec)$, to consider the transmission of signals over neural pathways [62, 86, 90, 91, 101–109, 111, 113]. A muscle activation dynamics (MAD) model, reflecting underlying biochemical processes such as calcium kinetics and cross-bridge dynamics, was used in 18 studies [42, 86, 90, 91, 95–98, 100, 103–109, 111, 113], causing a smoothened joint torque response to muscle excitation. Seven studies reported a reflex sensitivity measure using the Angle of Catch *AoC  (deg)*, not depicted in Fig. 5. This is identified as the point of peak torque increase [56, 63, 68, 70, 84] or phasic EMG rise [44, 86].

#### Intrinsic neural drive

Seven studies identified the activation of muscles through intrinsic neural drive as an increase in intrinsic resistance parameters, i.e., joint stiffness or damping (*K* or *B*) [87, 99, 101, 102, 110, 111, 113]. Nine studies quantified a resistance torque ($T_{INT-N}$), independent of rotation or velocity and resulting from intrinsic neural drive, filtered through MAD. This intrinsic drive can be voluntary during active task instruction or involuntary, due to sustained drive, synergistic, or co-contraction activation in UMNS. Three studies used the measured tonic EMG (*EMG-T, (mV)*) [99, 104, 110], a constant level independent of perturbation, to quantify intrinsic neural drive.

#### Rate of occurrence across studies

The bar chart in Fig. 5 shows the number of occurrences for each parameter. Studies commonly considered the reflexive neural drive and mechanical properties, while the influence of intrinsic neural drive drive on joint neuromechanics gained limited attention. Regarding the mechanical and spinal reflex level, respectively, joint stiffness (*K*) and the total reflex torque ($T_{REF}$) are observed more often than mechanical damping (*B* or $T_B$), and the parameterization of reflex resistance (using *Δ t*, $k_p$, and $k_v$).

### Input–output analysis - parameter value extraction

**Fig. 6:**
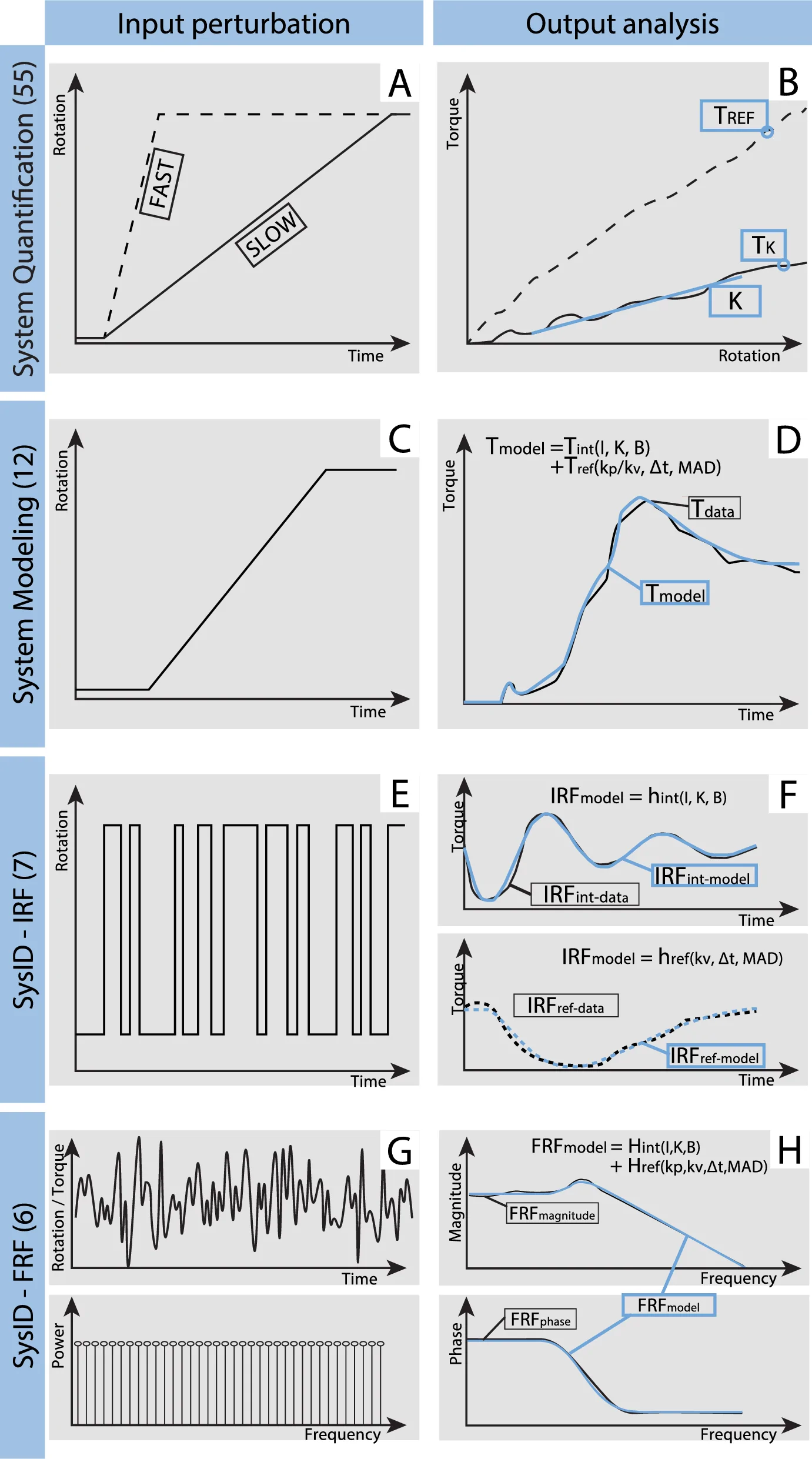
The four found analysis methods to define the neuromechanical parameters of Fig. 5: System Quantification (**A** and **B**), System Modeling (**C** and **D**), System Identification - IRF (**E** and **F**), and System Identification - FRF (**G** and **H**). Raw input–output data in black, parameter value extraction in blue. Written formulas indicate used parametric models. Occurrence counts (in brackets) exceed 74, as some studies performed multiple analyses. Images partially obtained from [45, 98, 106, 87]. *MAD =* muscle activation dynamics, *SysID* = system identification, *IRF =* impulse response function, *FRF =* frequency response function, *h* and *H* are IRF and FRF models, *int =* intrinsic, *ref =* reflexive

Four categories of analysis methods (Fig. 6) were identified in the included studies, based on those proposed by Westwick and Kearney [116]. Their commonality lies in using the provided joint perturbation as input and the measured response as output to extract values for a set of parameters depicted in Fig. 5. The first category, ’System Quantification’, uses point measures or slope averages of the input and output signals to quantify system parameters. The second category of ’System Modeling’ makes use of a priori joint neuromechanical models, with parameter values experimentally fitted using input and output data. The third category, ’System Identification’, starts out from a different perspective. No a priori model is initially assumed, but stochastic correlations of input and output signals are used to build a nonparametric system description. The ’System Identification’ category is split into two, addressing the ’impulse response function’ (IRF) and ’frequency response function’ (FRF) approaches separately. Although system identification is often followed by fitting a parametric model to obtain interpretable parameters, studies that start with a nonparametric description were grouped under System Identification.

#### System quantification

A total of 55 studies used either the slope or point measures of the torque-angle trace to directly quantify joint neuromechanical parameters (Fig. 6A&B) [40–94]. This torque-angle trace results from joint resistance to a (cyclic) stretch imposed by the device. The majority (44/55) of the ’System Quantification’ studies used multiple stretch velocities (Slow = *<20  deg/s*, Med. = *20-100  deg/s*, Fast = *>100  deg/s*, see Supplementary Material 2 for details) to separate different neuromechanical contributions [40–43, 45, 46, 50–53, 56–62, 64–69, 71–76, 80–94]. A slow stretch was expected to reveal the mechanical stiffness *K* or stiffness torque $T_K$, and a faster stretch to reveal the resistance as a result of a velocity-responsive reflex ($T_{REF}$).

To isolate the reflexive torque in the fast-stretch resistance, 38 studies corrected for passive mechanical contributions to fast-stretch resistance by subtracting slow-stretch or static passive stiffness, labeling the remainder as the phasic reflex component [40–43, 45, 46, 50, 52, 53, 57–62, 64–68, 71, 71, 72, 72–76, 80, 82–85, 90–94]. In addition, 17 studies attempted to also separate the velocity-responsive spinal reflex from velocity-responsive mechanical damping (*B*). Most studies achieved this through identifying $T_B$ or *B* at the onset of a fast stretch, ' reflex-free' due to neural time-delay, and using that to correct $T_{REF}$ later in the fast stretch [40–43, 52, 53, 60, 61, 64, 65, 73, 75, 76, 80, 90, 91, 94]. Another approach by Kamper et al. quantified the velocity-dependent resistance of the 'reflex-free' non-paretic limb as damping, using that to correct $T_{REF}$ of the paretic limb [62].

Ten studies in the System Quantification category did not perform this separation of passive mechanical resistance from neural reflex torques via slow and fast stretches [44, 47, 49, 54, 55, 63, 70, 77–79]. Instead, they quantified total resistance torque $T_{TOT}$ and inferred possible neural origin through comparison with another neural reflex measure, such as *EMG-P* [47, 49, 54, 55, 63, 77–79] or *AoC* [44, 63, 70].

With most included studies in this category focusing on the separation of passive mechanical and reflexive resistance, only a few assessed the intrinsic neural drive contribution to joint neuromechanics. Three studies assessed a voluntary generation of $T_{INT-N}$ during a move task, separating it from involuntary reflexive $T_{REF}$ by using its relative timing and direction with respect to the joint rotation [59, 72, 83]. One study assessed an involuntary ’resting’ $T_{INT-N}$ during a relax task, which could be separated from mechanical stiffness due to its independence of joint rotation angle [75].

#### System modeling

In the 12 System Modeling studies, the torque-angle data was used to fit neuromechanical parameters of an a priori established model (Fig. 6C&D) [42, 62, 90, 91, 95–102]. The fitting of parameter values from perturbation experiments was performed using nonlinear optimization algorithms, minimizing a criterion function such as depicted in Eq. 1:

1 $$\begin{aligned} L(p) = \sum |T_{meas}(t) - T_{mod}(\theta , \dot{\theta }, \ddot{\theta }, EMG, p)| \end{aligned}$$

where *p* is the total set of model parameters, $T_{meas}$ and $T_{mod}$ are the measured and modeled torque response to a perturbation, and *θ *, $\dot{\theta }$, $\ddot{\theta }$ are the perturbation angle, velocity and acceleration respectively. Most studies (10/12) extracted all neuromechanical model parameters within a single fit, on datasets containing both low and high perturbation velocity components [42, 62, 95–98, 100–102]. For this, different multi-velocity input perturbations were used, ranging from ramp-and-hold stretches of multiple velocities [42, 95–98, 100], to pulse perturbations [62, 99], or white noise perturbations [101, 102].

All but one [99] study included a passive mechanical and a reflexive component in their model. The used mechanical model components consisted of a stiffness *K* in all, and a damping *B* in some [62, 90, 91, 95, 98, 99, 101, 102] of the studies. These were modeled as linear parameters [62, 99, 101, 102], or as parts of nonlinear physiological MTU models consisting of exponential stiffness, damping, or muscle slack length [42, 90, 91, 95–98, 100]. The reflex model to obtain $T_{REF}$ consisted of some motor unit (MU) excitation level fed into MAD. In five studies, this MU excitation was calculated through the modeling of muscle spindles and the motor neuron pools, using $k_p$, $k_v$, and *Δ t* to convert rotation and velocity to MU excitation [62, 90, 91, 101, 102]. Alternatively, the MU excitation was directly measured using the EMG signal as a model input, removing the need to model reflexive feedback, as this is implicitly present in the EMG signal [42, 95–98, 100]. The subsequent MAD, to convert excitation to torque, ranged from a direct feed-through [62, 101, 102] or a linear filter [90, 91], to nonlinear Hill-type muscle models showing force-length and force-velocity relationships [42, 95–98, 100]. Only five of the studies in this category accounted for a torque unrelated to rotation or velocity, which could be related to intrinsic neural drive torque $T_{INT-N}$ [62, 98, 100–102]. This $T_{INT-N}$ was modeled by feeding a tonic EMG signal through an MAD model [100], or by directly measuring a constant tonic offset torque during perturbation trials [62, 98, 101, 102].

#### System identification - impulse response

The included System Identification approaches used stochastic correlations between input and output signals to form a nonparametric system description, prior to model fitting. Seven of the included studies performed such an analysis in the time domain, using auto- and cross-correlations to obtain the IRF (Fig. 6E&F) [103–109]. Correlations of input and output data effectively average over time, reducing the influence of noise on the nonparametric IRF.

The included studies utilized pseudorandom binary sequence (PRBS) pulse train perturbations as input for the IRF analysis. During the small-amplitude pulses, the maximum speed was around $\sim 115 \, deg/s$, and the sharp transitions resulted in a wide frequency range of perturbations (Bandwidth *≃ 75*), allowing full capture of joint dynamics. The hold periods, with a random duration of 150 to 1050 ms, reduced the average perturbation velocity to $\sim 17\, rad/s$.

Using the input–output data, all studies in this category employed the same approach to calculating two IRFs: one describing nonparametric intrinsic resistance and the other describing nonparametric reflexive resistance. The intrinsic IRF between rotation and torque was calculated using the initial period after a perturbation, with a fixed length shorter than the expected reflex delay *(≤ 40 ms)* [104]. The reflexive IRF was calculated over the full stretch response, using joint velocity as input and torque as output, capturing the velocity-dependent component of the reflex.

Subsequent to the nonparametric formation of an intrinsic and a reflexive IRF, all studies fitted the same parametric model (Fig. 6F) to the data. The intrinsic model consisted of the mechanical properties inertia *I*, stiffness *K*, and damping *B*, and the phasic reflex model consisted of reflex velocity-gain $k_v$, time-delay *Δ t*, and an MAD filter. Intrinsic neural drive was only considered in one of the studies from the System Identification - IRF category, where *EMG-T* was quantified to assess its influence on different neuromechanical parameters [104].

#### System identification - frequency response

Six included studies performed a nonparametric system description in the frequency domain [86, 87, 110–113] (Fig. 6G&H). This was done by dividing the auto- and cross-spectra of the input and output to obtain the FRF. The nonparametric FRF consists of a phase, which shows the time delay of the reflex as a phase shift, and a gain, which represents rotation-, velocity-, or even acceleration-dependent resistance across different frequency bands. All studies in this category subsequently extracted neuromechanical parameter values from the nonparametric FRF features. Moreover, three studies also reported an FRF between joint rotation and measured EMG signal, which was used to refine or determine reflex parameter estimates [110, 111, 113].

The used parametric models in the frequency domain were similar to those used in the IRF analysis, consisting of a mechanical *K*, *B*, and *I*, and a reflexive component with $k_v$, *Δ t*, and second-order MAD [86, 111, 113]. As an alternative or addition to $k_v$, the rotation-responsive reflex component $k_p$ was investigated in two studies [110, 111]. While used as the main reflex parameter in Burne et al [110], $k_p$ was shown to reduce model fit performance by Meskers et al. [111]. Even the addition of reflexive force feedback $k_f$ to the model was studied in a single record, accounting for a large part of the reflex resistance [113].

Alternatively, two studies adopted a simpler parameter set, fitting only a lumped *K*, *B*, and *I* to the system’s frequency response [87, 112]. Distinguishing reflexive and passive mechanical origin was then performed through additional System Quantification using slow and fast stretches [87], or by qualitatively differentiating mechanical behavior from reflex involvement, which the authors interpreted through variations in *K* and *B* [112].

The studies used periodic perturbations for the FRF analysis. This ranged from single sinusoids repeated at different frequencies [112], to combinations of different frequency components in a single signal. The latter was executed using a stochastic white noise [110] signal, or a quasi-stochastic random phase multisine consisting of specific frequencies added together with random respective phase shifts [86, 87, 111, 113]. Most studies limited the perturbation amplitude to satisfy the assumption of joint linearity, and the bandwidth (with a reported range from *0.5 - 50 Hz*) was chosen to cover relevant joint dynamics, but not suppress reflex responses, as shown in [111].

### Reliabiliy and validity

The most observed reliability assessments were the intraclass correlation coefficient (ICC, presented 26 times over 12 studies) [40, 48, 52, 69, 76, 82, 86, 87, 89, 95, 100, 112] and minimal detectable change (MDC, presented 19 times over nine studies) [40, 52, 82, 86, 87, 89, 96, 100, 112]. In Table 2, these results are summarized over studies for the different parameter groups. Complete reliability data can be found in Supplementary Material 3. Ranges for each parameter and assessment method are provided, with the MDC normalized to the mean or median of that parameter reported. As only one study reported reliability for $T_{INT-N}$ and none for *EMG-T*, these parameters were omitted from Table 2.

**Table 2:** Overview of the ranges of reliability test outcomes and the number of test-appearances (in brackets) per analysis type (Fig. 6) and parameter group (Fig. 5)

Analysis type	Test type	Stiffness $T_K/K$	Damping $T_B/B$	Reflex $T_{REF}/k_v/k_p/EMG-P$
System quantification	ICC	0.86–0.97 (7)	0.61–0.9 (3)	0.85–0.97 (7)
	MDC	70%–100% (4)	90%–139% (2)	30%–89% (4)
System modeling	ICC	0.93–0.99 (2)	N.A.	0.8–0.83 (2)
	MDC	9%–130% (2)	N.A.	89%–174% (2)
SysID IRF	ICC	N.A.	N.A.	N.A.
	MDC	N.A.	N.A.	N.A.
SysID FRF	ICC	0.50–0.79 (2)	0.59–0.94 (2)	0.52 (1)
	MDC	29%–87% (2)	20%–145% (2)	947% (1)

Refer to Supplementary Material 3 for individual study details

ICC = intraclass correlation coefficient, MDC = minimal detectable change, IRF = impulse response function, FRF = frequency response function

$^a$ All MDCs were normalized to the mean or median values of the respective parameter in each study

**Table 3:** Overview of validity test outcomes and the number of test-appearances (in brackets) per analysis method (Fig. 6) and parameter group (Fig. 5)

Analysis type	Test type	Stiffness $T_K/K$	Damping $T_B/B$	Reflex $T_{REF}/k_v/k_p/EMG-P$
System Quantification	Paretic vs. control$^a$	60% (15)	43% (7)	92% (26)
	Clinical scale$^b$	38% (8)	25% (4)	68% (19)
System modeling	Paretic vs. control$^a$	89% (9)	29% (7)	78% (9)
	Clinical scale$^b$	43% (7)	0% (3)	60% (5)
SysID IRF	Paretic vs. control$^a$	80% (5)	0% (4)	100% (5)
	Clinical scale$^b$	0% (1)	N.A.	0% (1)
SysID FRF	Paretic vs. control$^a$	80% (5)	66% (3)	57% (7)
	Clinical scale$^b$	50% (2)	0% (2)	40% (5)

Refer to Supplementary Material 4 for individual study details. IRF = impulse response function, FRF = frequency response function

$^a$ The percentage of studies finding results in line with expected clinical effects (hyperresistance or paresis)

$^b$ The percentage of studies finding results in line with manual clinical assessments

The two most commonly used validity tests in the included studies were: (1) accordance with clinical scales (presented 57 times for different parameters over 32 studies) [40, 42–46, 55, 57, 62–66, 70, 75–78, 80, 81, 84–86, 89, 95, 96, 98–100, 103, 112, 113], and (2) comparison between paretic joints and healthy controls (presented 102 times over 44 studies) [40, 41, 48–50, 54, 55, 59, 62, 62–66, 69, 72, 75–78, 81, 83, 85–87, 89, 90, 94–96, 98–107, 110–113]. The latter checked for consistency with the expected outcomes for UMNS: increased parameter values for hyperresistance or decreased parameter values for paresis assessment. These two most observed validity assessments were bundled over all studies for different parameter groups in Table 3. Complete validity data can be found in Supplementary Material 4. Other validity tests that were performed ranged from the comparison of two quantifications of the same construct, i.e., $T_{REF}$ and *EMG-P* (presented eight times) [46, 49, 58, 65, 76, 77, 79, 81], to showing the expected parameter response to treatments (presented 12 times) [43, 53, 56, 60, 61, 73, 90, 100, 108, 109].

## Discussion

This systematic review of the literature explored the diversity of I-NMA approaches, using controlled joint perturbations, in UMNS. We compared approaches by identifying and linking their experimental protocols, input–output analyses to quantify neuromechanical parameters, and reported parameter reliability and validity.

Overall, most studies assessed joint neuromechanics with participants relaxed, focused on a single joint, and applied stretches over (almost) the full (p)ROM. Less attention was given to studying multi-joint coupling, or assessments during active resistance to a perturbation, voluntary activation, or voluntary movement. Input–output analyses primarily focused on quantifying passive mechanical stiffness and reflex torque parameters, with intrinsic neural drive contributions often not quantified. Parameters were most often quantified using joint stretches and point measures or slope averages of the resistance torque (’System Quantification’). However, more advanced analytical approaches, such as System Modeling and System Identification (IRF or FRF), were also adopted. System Modelling uses a priori joint neuromechanical models with parameters fitted to measured input–output data. In contrast, System Identification derives a nonparametric system description from stochastic correlations between input and output signals. Although the System Modeling and Identification approaches offer methodologies to more thoroughly separate different constructs, the proof for increased validity of quantification (Table 3) remains lacking, and reduced reliability of the parameters compared to the lumped System Quantification approach is a concern (Table 2).

### Experimental protocol

The most common choice of experimental protocol is a relax task in OL human-device interaction, and full (p)ROM joint stretches. This choice of experimental protocol focuses I-NMA on the evaluation of involuntary hyperresistance of a single joint. Although this protocol is straightforward to implement and achievable for most patient groups, the information obtained could overlook vital elements.

First, the neuromechanics of a single joint cannot be seen as a separate entity from the rest of the limb, or even the body. All included studies assessing multi-joint neuromechanics found some form of inter-joint or inter-limb coupling, on either the mechanical or neural (intrinsic or reflexive drive) level [57, 59, 69, 71, 72, 83, 87, 92, 93, 100]. In UMNS, inter-joint coupling becomes highly relevant when pathologically affected through synergies [31]. Diagnosing these pathological synergies and separating them from other phenomena which limit active movement (e.g., MTU stiffness, hyperreflexia, and paresis) would be valuable [34], but comes at the cost of more complex and expensive devices for multi-joint assessment [117].

Second, changes in joint neuromechanics in UMNS were shown to manifest differently in relaxed and active participant tasks [59, 72, 83, 99, 104, 110]. This difference arises due to UMNS manifesting both as hyperresistance (increased resistance when attempting to relax) and paresis (reduced resistance when actively engaged), as shown in BOX 1. This underscores the importance of also assessing participants while they are active, such as during ’position’, ’activation’, or ’move’ tasks.

Likely, some studies on active task performance in UMNS were excluded for lacking a controlled perturbation or input–output analysis needed for quantification of neuromechanical parameters. Instead, these analyses rely on general performance metrics (e.g., max. force, reaction time, reach velocity). CL perturbation assessments are a potential technical approach for facilitating active tasks, while also allowing for neuromechanical quantification using input–output relationships. During CL assessments, the human is not fully subject to the perturbations, but can influence the combined movement of the limb and device [5, 118]. Tweaking the virtual environment in a CL assessment can be used to assess the changes in motor control strategy when virtual mass, damping, or stiffness are added [86, 111, 119].

For assessment during active movement, the OL and CL set-ups can even be combined within a single protocol. Here, free voluntary movement is allowed in CL interaction between human and device, and a brief switch to OL control with a rotational perturbation allows for joint manipulation at highly specific velocities and accelerations [120].

Lastly, most studies measured over (almost) the full (p)ROM. Yet, joint neuromechanics are inherently nonlinear and can change substantially over the (p)ROM [121]. The studies that perform perturbations over a large section of the (p)ROM are thus forced to a linear simplification, or a more complex nonlinear model. The small and linear range amplitudes of most used periodic or (pseudo)stochastic perturbations are not sensitive to these nonlinearities [115], but thus also do not allow for their quantification. Repeated small-amplitude perturbations at different positions of the joint could provide a solution, explicitly assessing the changes in joint neuromechanics with respect to joint posture. This analysis is performed in nine of the included studies, which indeed show a clear change in parameters over the (p)ROM [62, 99, 103–109]. However, this approach is time-consuming and could cause fatigue in the assessed patients or research participants.

Furthermore, the repetition of small-amplitude perturbations at different joint angles accounts only for joint-angle-related nonlinearity and does not assess dynamic state changes, such as the ’spastic catch’ phenomenon. The latter can occur in UMNS patients during a brisk stretch movement, when the joint switches to a state of increased tone [114]. The state of heightened tone is possibly due to PICs, regeneratively activated currents in spinal motorneurons that cause sustained firing after stretch reflex excitation [32, 33, 58]. A few included studies report such persistent or emerging resistance after a fast stretch [48, 58, 92–94].

To efficiently and accurately capture joint-angle nonlinearity and dynamic state changes during neuromechanical assessment, time-varying system identification could provide a solution. These identification techniques enable a joint model that adapts its properties over time [122], often using ensemble averaging of multiple responses that share the same non-stationarities [121]. Research in healthy participants and simulations shows promise for reliably quantifying time-varying joint behavior [123], but further work is needed to apply this to UMNS joints, which exhibit greater nonlinearities [106] and higher variability across trials [27, 124].

### Input–output analysis

#### Separating mechanical and reflexive contributions

Each of the four input–output analysis methods (System Quantification, System Modeling, and System Identification (IRF or FRF)) used a different approach to separate the mechanical and reflexive resistance. Most System Quantification studies assume a purely rotation-responsive mechanical stiffness and a velocity-responsive reflex torque. This velocity-based separation of reflexive and mechanical contributions rests on the assumption that resistance to slow and fast stretches will be exclusively of passive mechanical or reflexive origin, respectively. However, the absence of reflexive resistance to slow stretches is not undisputed, as phasic reflex EMG has been reported during slow stretches in some included studies [81, 98]. Furthermore, the ignored joint damping (*B* or $T_B$) results in a velocity-dependent passive mechanical resistance, which clouds the quantification of the reflexive resistance during a fast stretch. A commonly described workaround for the latter is to separate the immediate velocity-responsive torque, attributed to damping, from the delayed onset of the phasic reflex. While this approach allows some distinction, it typically relies on just two-point measures taken near the start and end of the fast stretch, neglecting the full temporal dynamics of these mechanisms. Nonlinear increases in joint damping during stretch may be misattributed to reflex activity, and errors in damping quantification can propagate into reflex estimates. This yields only a coarse separation and less accurate estimates of both contributions [42, 90, 91].

Studies in the ’System Modeling’ and ’System Identification’ category separate passive mechanical and reflexive contributions through explicitly incorporating the reflex time-delay (*Δ t*) in their models. In the ’System Identification’ approaches, the time delay even explicitly arises in the nonparametric neuromechanical description, prior to model fitting. In the FRF approach, the time delay presents itself as a phase shift, while in the IRF approach, a separate and time-delayed reflex IRF is formed. As the time delay emerges directly in the nonparametric FRF or IRF representation, the subsequent parametric modeling of time-delayed reflex becomes less sensitive to structural model mismatch and measurement noise [105, 121].

Using a time-delay in the spinal reflex model enables parameterization of reflexive rotation- ($k_p$), velocity- ($k_v$), and even acceleration- ($k_a$) or torque- ($k_f$) responsive components, without overlapping with passive mechanical constructs responding to the same kinematic quantities (*K*, *B*, *I*). Regarding the reflexive parameters used in the ’System Modeling’ and ’System Identification’ studies, there seems to be a general consensus in the literature that the reflex contains a velocity-responsive component $k_v$. However, while there is general agreement that the length-dependent type II-pathway ($k_p$) and the force-dependent Ib-pathway ($k_f$) are also subject to change in UMNS [4], their actual estimated contributions to joint neuromechanics are ambiguous [101, 102, 111, 113]. Research in healthy participants shows significant contributions of both $k_p$ and $k_f$ [125], which are dependent on task-instructions to the human [126]. This task-dependent contribution of different model components warrants further exploration in a UMNS population with altered motor control [127]. Especially when expanding experimental protocols towards more active tasks, the role of Ib-inhibition (represented by $k_f$) could be further explored [126].

Besides velocity-based or time-delay-based separation, EMG measurements can be used to distinguish mechanical and reflexive resistance. These EMG measurements were used as additional data to torque and rotation in 7 out of 12 studies in the ’System Modeling’ category and in four ’System Identification’ studies, using the EMG signal as input to their models to refine or determine $T_{REF}$. Alternatively, in 24 studies, an independent *EMG-P* parameter was quantified using the phasic EMG signal, to compare to measured resistance torques and infer whether these arose due to mechanical or reflexive contributions. While likely effective for separating reflexive and mechanical contributions, EMG measurements are known to increase variability and reduce the reliability of the assessment [42, 128]. Furthermore, complementary EMG measurements require more assessment time, reducing their suitability for a clinical setting.

#### Intrinsic neural drive contribution

Contrary to the quantification of mechanical properties and reflexive resistance, the quantification of intrinsic neural drive contributions *EMG-T* (n=3), $T_{INT-N}$ (n=9), and the influence of intrinsic neural drive on *K* (n=7) and *B* (n=6) receive limited attention in the included studies. One possible explanation is the limited use of active task instructions in included studies, as these facilitate the quantification of intrinsic neural drive, thought to originate predominantly from cortical pathways. The intrinsic neural drive is, however, of great relevance in UMNS joint neuromechanics [110]. Not only during active engagement or movement, but also in a passive state (’relaxed’), due to involuntary sustained muscle drive or co-contraction contributions to hyperresistance.

Another explanation for the reduced attention for intrinsic neural drive is that separating this contribution is less straightforward. While the time-delay or velocity-dependence can be useful tools for separating the reflexive from mechanical resistance, the influence of intrinsic neural drive cannot be isolated through these methods due to its intrinsic nature. For separating mechanical properties from intrinsic neural drive contributions, an analysis of EMG signal (*EMG-T*) was thus used in some studies, forming a viable solution. In future research, EMG analysis could be extended further to distinguish the origin of neural drive. Using frequency-domain EMG coherence, shared oscillatory activity between motor units across different frequency bands can be used to infer their upstream driving source to be either afferent or corticospinal [129–131]. EMG signal could also be combined with electroencephalography (EEG) to explore corticomuscular coherence and to verify whether observed changes in neural drive are indeed of descending corticospinal origin [132].

Alternatively, if a purely kinetic approach is sought, the torque independent of rotation or velocity, $T_{INT-N}$, could be further explored for quantifying intrinsic neural drive. However, intrinsic neural co-contraction will not result in such a $T_{INT-N}$ while still influencing joint stiffness and damping [121], which warrants further research on how to robustly separate intrinsic neural drive contributions from passive mechanical properties without the use of EMG.

#### Input perturbation

The applied perturbation must span a sufficiently broad range of velocities to properly separate the rotation-dependent (e.g., stiffness), velocity-dependent (e.g., reflexive), and acceleration-dependent (e.g., inertial) contributions to joint neuromechanics. This is supported by the included literature, as almost all studies use perturbations consisting of multiple velocity components, with the few exceptions relying on EMG signal for construct separation (see Supplementary Material 2). Most studies achieve different stretch velocities by using low- and high-velocity stretches across (almost) the full (p)ROM. However, (pseudo)stochastic perturbations, used in several System Modeling and System Identification approaches, offer greater excitation richness and do so more efficiently, as they span a broad range of velocities and frequencies within a single experiment. Some considerations arise in the design of these (pseudo)stochastic perturbations to optimally quantify joint neuromechanics.

First, to describe full system dynamics, the frequency content of the perturbation should extend beyond the joint’s eigenfrequency, where a mechanical response is still expected [121]. However, while most included studies use a high-velocity stretch to elicit a reflex, in the case of (pseudo)stochastic perturbations, excessively high average velocities or perturbation frequencies are known to suppress the reflex response [101, 119, 133]. Second, the input signal amplitude must remain limited to enable a linear analysis [134], which inevitably also limits the signal-to-noise ratio of the recorded signals [134]. Lastly, perturbation signals require unpredictability to eliminate the contribution of interfering feedforward voluntary responses from the human.

Stochastic perturbation signals, such as white noise, offer a broad frequency spectrum and complete unpredictability. Pseudo-stochastic perturbations, such as a PRBS and multisine perturbations, offer the added advantage of periodicity for averaging in analysis, while remaining unpredictable due to a randomized interval or phase component [121]. Furthermore, these perturbations are customizable in their frequency and velocity content, and can be optimized for high signal power within the maximal amplitude. Balancing the described perturbation trade-offs, tailored to the neuromechanical construct under measurement, is an active field of research [119, 134–136]. For advances in input perturbation design and subsequent parametric or nonparametric analysis, inspiration could be drawn from other research fields, such as control engineering [137].

### Clinical value

#### Validity

The results presented in Table 3 show that when comparing parameter values to clinical scales such as the MAS, MTS, or the Fugl-Meyer Assessment, the validity is low, as indicated by a low percentage of ’affirmative outcomes’ for this type of assessment. A possible explanation for the lack of correlation is the comparison of an ordinal clinical scale with a continuous I-NMA variable. More importantly, a lack of correlation between I-NMA and a clinical test would leave it unclear which measure is more valid [103]. Currently, there is no true ’gold standard’ for separating neuromechanical constructs in clinical tests, as many existing methods are criticized for this very limitation [13, 15–17].

As an alternative, included records test whether I-NMA parameters differ between paretic and control joints as theoretically expected, an approach that showed higher and more consistent validity. However, even for this test, reduced validity scores for the joint damping parameters *B* and $T_B$ are evident in Table 3. This could be explained by the discussed difficulty in separating this construct from reflexive resistance, which hampers quantification.

Although ‘System Modeling’ and ‘System Identification’ methods use more detailed neuromechanical models to enable more thorough separation of mechanical and reflexive contributions, the validity assessment in Table 3 shows no clear improvement over the ‘System Quantification’ approach. Further research, using different techniques (EEG, EMG) for external validation or longitudinal studies to assess intervention effects, is needed to investigate whether these more detailed models indeed provide more valid clinical information.

#### Reliability

The reliability results in Table 2 indicate lower reliability ICC scores for the more complex ‘System Modeling’ and ‘System Identification’ approaches compared to ‘System Quantification’. This effect is particularly notable for System Identification approaches, despite their use of time- or ensemble-averaging to mitigate noise. Increased model complexity may have reduced the reliability of individual parameters for these methods, as also reflected by relatively high standard errors of measurement reported for parameters in complex models [98, 100]. While detailed models may be valuable in research settings, clinical application requires both reliability and validity. This requires balancing adequate construct separation against over-parameterization, which would reduce reliability.

Besides the model and analysis, the experimental setup and its consistency have a large influence on the reliability. Uncorrected, or unknown, perturbator dynamics, as well as inconsistent attachment and alignment between joint and perturbator, can result in error propagation and inter-experiment differences [17, 65, 68, 76, 121]. Assessing the impact of experimental uncertainties and developing noise-reducing analysis strategies to ensure consistent quantifications can improve the reliability of I-NMA. Next to noise in the analysis and inconsistencies in the experimental setup, variations in human behavior and performance also reduce the reliability of the quantified neuromechanical parameters [112]. This variation is expected to be greater in UMNS patients than in healthy individuals, thereby increasing measurement uncertainty in a clinical setting. For example, the number of previous stretches [27, 124], physiological stress [124], fatigue [121], and even outside temperature [33] have been shown to affect joint neuromechanical parameters in UMNS patients.

For treatment evaluation and prediction, I-NMA outcomes require not only adequate relative reliability (high ICC) but also good absolute reliability (low MDC) to identify changes within an individual. Table 2 shows that even with a high ICC, the MDC score can yield less positive results. Most reported MDC-values were around 100% of the median or mean, indicating that the smallest clinical difference that can be observed is of the same order of magnitude as the total found parameter value. This raises concerns about the sensitivity of I-NMA to detect clinically meaningful changes at the individual level.

In total, only 14 out of 74 studies reported a reliability score for I-NMA. This is a key limitation in the literature, which focuses on developing new techniques, protocols, and algorithms rather than evaluating their reliability. As reliable assessment is a prerequisite for clinical value, further research should be conducted on the reliability of I-NMA.

#### Clinical impact

Advancing towards greater clinical value of I-NMA may yield substantial benefits, given the considerable investments in pharmaceutical treatments and physical therapy targeting altered joint neuromechanics in UMNS. Illustratively, annual rehabilitation costs are estimated between *∼ 45 - 60* [138] thousand USD per patient, of which *∼ 5* thousand USD goes towards pharmaceutical anti-spasticity treatment [139] and another *∼ 2--7* thousand towards physical therapy [140]. In light of the variable individual responses to these treatments [20, 141], I-NMA has the potential to support a valuable shift towards more personalized treatment selection and evaluation. A baseline I-NMA could quantify neural and mechanical contributions to abnormal joint resistance or reduced joint function, thereby guiding treatment selection. A follow-up assessment could then evaluate treatment effects and inform necessary adjustments. To realise this potential, the improved objectivity and resolution of I-NMA over current clinical assessments must be complemented by low MDC values. In addition, robust longitudinal evidence is needed to further assess its ability to detect treatment effects and disease progression, or to serve as a predictive biomarker. However, such studies remain scarce in the current literature, consistent with a previous review by van der Velden et al., which highlighted the limited evidence for the clinical benefit of I-NMA [26].

### Recommendations

Based on the reviewed studies and the presented discussion, a few ’best practices’ and recommendations are defined:

1. Include active assessments. Assess both passive and active conditions to quantify hyperresistance and paresis within a single protocol.
2. Quantify separate UMNS contributions. Use multi-velocity perturbations and analysis methods that separate mechanical, reflexive neural, and intrinsic neural contributions. The latter may require further model development or additional signals such as EMG or EEG.
3. Improve clinical value. Evaluate whether the more advanced neuromechanical models improve construct separation validity, and perform longitudinal studies to establish diagnostic and prognostic value. Further assess the reliability of I-NMA outcomes and seek improvement through protocol consistency and noise-reducing analyses.
4. Improve clinical applicability. Extend assessments to functional movements by developing multi-joint and closed-loop perturbation devices, and methods that capture time-varying and nonlinear neuromechanics.

### Limitations

#### Excluded research fields

With a focus on separate but concurrent neural and mechanical quantification, studies solely quantifying a neural measure (e.g., only using EMG measurements, H-reflex assessment, or tendon taps), or articles solely quantifying passive mechanical properties (e.g., using only very slow stretches) were excluded from the review. This could have led to the exclusion of possible techniques for isolating a specific component of joint neuromechanics. Furthermore, articles that utilized manual perturbation with sensor-based data analysis were excluded, as manual perturbations are less consistent than controlled ones, which can influence I-NMA outcomes [67, 142]. This type of study may have provided an interesting perspective on simpler approaches to improved I-NMA in UMNS, with high clinical applicability [143]. Lastly, studies assessing unperturbed voluntary movement were excluded, as these inherently reflect combined effects of cognitive control, muscle activation, and passive tissue properties, limiting construct separation. These analyses could complement the presented results, enabling a functional assessment of neuromechanics during movement [24].

#### Possible bias

We excluded papers based on specific bibliographic and study characteristics, including the number of included subjects, language, and publication type. While this could lead to certain innovative and exploratory techniques being excluded, it is expected that successful I-NMA approaches will eventually be executed in larger study populations, resulting in journal publications. Excluding all articles published before 2005 could have overlooked older I-NMA approaches. However, relevant approaches are expected to be present as building blocks for the work included in this review. For a comprehensive review of similar techniques published before 2005, the reader is directed towards Wood et al., 2005 [21]. A quality assessment was omitted due to the qualitative nature of the review, the heterogeneity of methodologies, and the inconsistent evaluation of I-NMA outcome reliability and validity in the studies, which make direct comparisons challenging.

## Conclusion

This systematic literature review presents an overview of 74 studies that used a controlled perturbation and input–output analysis to quantify neuromechanical parameters in different instrumented neuromechanical assessment (I-NMA) approaches. We examined how choices in experimental protocol and analysis affect the reliable and valid quantification of mechanical and neural (intrinsic and reflexive) contributions to joint neuromechanics in upper motor neuron syndrome (UMNS). The included literature focused heavily on assessment solely in relaxed rather than active conditions (63/74 studies), and the quantification of mechanical stiffness from slow stretch and reflex torque from fast stretch resistance (55/74 studies). However, this separation is not straightforward: slow stretches may still elicit reflexes, while faster stretching can also alter mechanical resistance, meaning the two are not mutually exclusive. More advanced joint neuromechanical models that fit models to input–output relations while explicitly separating time-delayed reflexes from mechanical stiffness, damping, and inertia might improve the validity of separating UMNS constructs, but the current evidence does not yet provide proof for this. Furthermore, these more extensive assessments do show a reduced reliability in the included studies, indicating the need for further improvements. Reliability can be improved through consistency in the experimental protocol, optimal perturbation design, and improved parameter-fitting procedures in the presence of noisy data. Furthermore, clinical value should be enhanced by adopting setups to assess active ’move’ or ’position’ tasks to capture paresis, and by incorporating analyses that address nonlinearities, time-variance, and interjoint coupling in joint neuromechanics. Finally, further clinical evidence is needed to substantiate the potential benefits of I-NMA-guided UMNS rehabilitation treatments.

## Data Availability

No datasets were generated or analysed during the current study.

## Supplemenatry information

Supplementary Material

## Abbreviations

ALS: Amyotrophic lateral sclerosis
CL: Closed loop
CP: Cerebral palsy
CVA: Cerebrovascular accident
EEG: Electroencephalography
EMG: Electromyography
FRF: Frequency response function
ICC: Intraclass correlation coefficient
I-NMA: Instrumented neuromechanical assessment
IRF: Impulse response function
MAD: Muscle activation dynamics
MAS: Modified Ashworth scale
MCP: Metacarpophalangeal
MDC: Minimal detectable change
MS: Multiple sclerosis
MTS: Modified Tardieu Scale
MTU: Muscle tendon unit
MU: Motor unit
OL: Open loop
PIC: Persistent inward current
(p)ROM: (Passive) range of motion
PRBS: Pseudorandom binary sequence
SCI: Spinal cord injury
TBI: Traumatic brain injury
UMNS: Upper motor neuron syndrome

## Footnotes

1 Hyperresistance is often clinically referred to as ’spasticity’. In this review, however, spasticity corresponds to ’hyperreflexia’, which is just one of the underlying components of hyperresistance.
