# Supplementary material for: Instrumented assessment of joint neuromechanics in upper motor neuron syndrome: a systematic review of methods, reliability, validity, and clinical relevance
Source: J Neuroeng Rehabil. 2026 Jul 29;23:309. doi: 10.1186/s12984-026-02108-1 (PMC13700493; doi:10.1186/s12984-026-02108-1)
Supplement: Supplementary file 1 [file 12984_2026_2108_MOESM1_ESM.pdf]

Supplementary Material

"Instrumented Assessment of Joint Neuromechanics in Upper Motor Neuron Syndrome - A Systematic Review of Methods, Reliability, Validity, and Clinical Relevance."

Karien ter Welle, Alfred C. Schouten, Winfred Mugge, Jonathan C. van Zanten, Erwin E.H. van Wegen, Arno H.A. Stienen, Carel G.M. Meskers, Mark van de Ruit

Supplementary Material 1 — Search terms for database search

Search terms were divided into 5 categories: Human Joint, Neuromechanics, Quantification, Controlled Perturbation, Upper Motor Nueron Synrome (UMNS). Synonyms in each category were combined with the OR-operator, categories were combined with the AND operator.

The * operator indicates trunction with free suffix. The "" operator indicates fixed appearance of two words in sequence. The NEAR/2 operator indicates that two words need to appear within 2 words distance from eachother. The operators were converted to equivalent operators per scientific database (SCOPUS or Web of Science).

Human Joint
Elbow; Knee; Wrist; Ankle; Shoulder; Hip; [Human NEAR/2 joint]

Neuromechanics
Impedance; Dynamic; Admittance; Damping; Stiffness; Intrins*; Reflex*; Resistance; Viscoelastic*; Neuromech*; Viscosity; Elasticity

Quantification
Identif*; Quantif*; Measure*; Assess*; Model*; Estimat*; Evaluat*

Controlled Perturbation
Robot*; Device; Manipulator; Instrumen*; [Controlled NEAR/2 torque]; [Controlled NEAR/2 stretch*]; [Constant NEAR/2 stretch*]; "Mechanical stretch*"; Perturb*; "Ramp and hold"; Ramp-and-hold; "Isokinetic dynamometer"

UMNS
Stroke; CP; MS; SCI; Hemipare*; UMNS; UMNL; Spastic*; Poststroke; Post-stroke; "Cerebral palsy"; "Spinal cord injury"; "Multiple sclerosis"; "Upper motor neuron syndrom*"; "Upper motor neuron lesion"

Supplementary Material 2 - Overview of all included studies and extracted data

Table 1: Overview of studies and extracted data. Reference numbers correspond to the reference list of the main text. In the column 'Joint': '&' = simultaneous manipulation/assessment, '+' = separate research lines for different joints, and brackets '()' = manipulated to assess effects on other joints, but not quantified itself. In UMNS the temporal phase is indicated between brackets: C = Chronic, (S)A = (Sub)Acute, and NS = Not specified, or both phases. In 'Perturbation': info between brackets indicate velocity (S = Slow ($\leq 20\frac{deg}{s}$), M = Medium ($20-100\frac{deg}{s}$), and F = Fast ($\geq 100\frac{deg}{s}$)) or amplitude/bandwidth. In 'Loop': OL = open-loop, CL = closed-loop. In 'EMG': Par. = EMG level used as separate parameter, Mod. = EMG data used in modeling, and Task = EMG used to check task-instruction compliance (e.g. fully relax). All parameters adhere to those used in Fig. 5 of the main text. Parameters are underlined if not only considered in the neuromechanics model, but also reported in the results section of the study.

Study	Joint	UMNS[a]	Perturbation	Loop	Task	EMG [b]	Quantified parameters[c]
**System Quantification**							
Andringa 2019 [40]	Wrist	CVA (C)	Stretch (S+F)	OL	Relax	-	I, $\underline{T_K}$, $\underline{T_B}$, $\underline{T_{REF}}$
Andringa 2020 [41]	Wrist	CVA ((S)A)	Stretch (S+F)	OL	Relax	-	I, $\underline{T_K}$, $\underline{T_B}$, $\underline{T_{REF}}$
Andringa 2021 [42]	Wrist	CVA (C)	Stretch (S+F)	OL	Relax	-	I, $\underline{T_K}$, T_B, $\underline{T_{REF}}$
Andringa 2022 [43]	Wrist	Stroke (C) + CP	Stretch (S+F)	OL	Relax	-	I, $\underline{T_K}$, $\underline{T_B}$, $\underline{T_{REF}}$
Annaswamy 2007 [44]	Ankle	TBI	Stretch (F)	OL	Relax	Mod.	$\underline{AoC}$, $\underline{T_{TOT}}$
Bhadane 2015 [45]	Elbow	CVA (C)	Stretch (S+M+F)	OL	Relax	-	T_K, $\underline{T_{REF}}$[d]
Boi 2024 [46]	Ankle	MS	Stretch (S+M+F)	OL	Relax	Par. Task	T_K, $\underline{T_{REF}}$, $\underline{EMG-P}$
Boudarham 2014 [47]	Knee	CVA (C)	Stretch (M+F)	OL	Relax	Par.	$\underline{T_{TOT}}$, $\underline{EMG-P}$
Centen 2017 [48]	Elbow	CVA (NS)	Constant torque stretches	OL	Relax	-	$\underline{T_K}$, $\underline{T_{REF}}$[f]
Damiano 2006 [49]	Knee	CP	Stretch (M+F)	OL	Relax	Par.	$\underline{T_{TOT}}$[d], $\underline{EMG-P}$
Dehno 2019 [50]	Wrist	CVA (SA)	Stretch (S+M+F)	OL	Relax	-	$\underline{T_K}$, $\underline{T_{REF}}$
Fang 2015 [51]	Ankle	SCI (C)	Cyclic stretch (S+M)[e]	OL	Relax	-	$\underline{T_K}$, $\underline{T_{REF}}$
Gäverth 2013 [52]	Wrist	CVA (C)	Stretch (S+F)	OL	Relax	-	I, $\underline{T_K}$, $\underline{T_B}$, $\underline{T_{REF}}$
Gäverth 2014 [53]	Wrist	CVA (C) + TBI	Stretch (S+F)	OL	Relax	-	I, $\underline{T_K}$, $\underline{T_B}$, $\underline{T_{REF}}$
Grey 2008 [54]	Ankle	MS + SCI	Stretch (F)	OL	Relax	Par. Task	$\underline{T_{TOT}}$, $\underline{EMG-P}$

Study	Joint	UMNS	Perturbation	Loop	Task	EMG	Parameters
Grippo 2011 [55]	Knee	SCI (NS)	Stretch (M+F)	OL	Relax	Par. Task	$\underline{T_{TOT}}$, $\underline{EMG-P}$
Hameau 2014 [56]	Knee	CVA (C)	Stretch (S+M+F)	OL	Relax	-	$\underline{K}$, $\underline{T_K}$, $\underline{T_{REF}}$, $\underline{AoC}$
Hoffmann 2009 [57]	MCP joint (& elbow & shoulder)	CVA (C)	Stretch (S+F)	OL	Relax	Par. Task	T_K, $\underline{T_{REF}}$, $\underline{EMG-P}$
Hornby 2006 [58]	Ankle	SCI (C)	Stretch (M+F)	OL	Relax	Par. Task	T_K, $\underline{T_{REF}}$, $\underline{EMG-P}$
Hyngstrom 2010 [59]	Hip & Knee & Ankle	CVA (C)	Cyclic stretch (S)[e]	OL	Relax + Move	Par.	I, T_K, $\underline{T_{REF}+T_{INT-N}}$[g], $\underline{EMG-P}$
Kachmar 2016 [60]	Wrist	CP	Stretch (S+F)	OL	Relax	-	I, T_K, T_B, $\underline{T_{REF}}$
Kachmar 2018 [61]	Wrist	CP	Stretch (S+F)	OL	Relax	-	I, $\underline{T_K}$, $\underline{T_B}$, $\underline{T_{REF}}$
Kamper 2006 [62]	MCP joint	CVA (C)	Stretch (S+F)	OL	Relax	Mod.	T_K, T_B, $\underline{T_{REF}}$, $\underline{\Delta t}$
Kim 2005 [63]	Ankle	CVA (NS)	Stretch (M+F)	OL	Relax	Par.	$\underline{T_{TOT}}$, $\underline{EMG-P}$, $\underline{AoC}$
Leng 2019 [64]	Wrist	CVA (NS)	Stretch (S+F)	OL	Relax	Task	I, $\underline{T_K}$, $\underline{T_B}$, $\underline{T_{REF}}$
Lindberg 2011 [65]	Wrist	CVA (C)	Stretch (S+F)	OL	Relax	Par.	I, $\underline{T_K}$, $\underline{T_B}$, $\underline{T_{REF}}$, $\underline{EMG-P}$
Lorentzen 2010 [66]	Ankle	CVA (NS) + MS + SCI (C)	Stretch (S+M+F)	OL	Relax	Par. Task	$\underline{T_K}$, $\underline{T_{REF}}$, $\underline{EMG-P}$
Lorentzen 2012 [67]	Ankle	MS + SCI (C)	Stretch (S+M+F)	OL	Relax	Par.	$\underline{T_K}$, $\underline{T_{REF}}$, $\underline{EMG-P}$
McPherson 2019 [68]	Elbow	CVA (C)	Stretch (S+M+F)	OL	Relax	Task	T_K, $\underline{T_{REF}}$[d], $\underline{AoC}$
Mizuno 2016 [69]	Ankle (& Knee)	CVA (SA)	Stretch (S+M)	OL	Relax	-	$\underline{T_K}$, $\underline{T_{REF}}$
Nam 2017 [70]	Elbow + Wrist	CVA (C)	Stretch (S+M+F)	OL	Relax	-	$\underline{T_{TOT}}$, $\underline{AoC}$
Onushko & Schmidt 2007 [71]	Hip & Knee & Ankle	SCI (C)	Cyclic stretch (S+M)[e]	OL	Relax	Par.	I, T_K, $\underline{T_{REF}}$, $\underline{EMG-P}$
Onushko 2010 [72]	Hip & Knee & Ankle	SCI (C)	Cyclic stretch (S+M)[e]	OL	Relax + Move	Par.	I, T_K, $\underline{T_{REF}+T_{INT-N}}$[g], $\underline{EMG-P}$
Palmcrantz 2020 [73]	Wrist	CVA (C)	Stretch (S+F)	OL	Relax	-	I, T_K, T_B, $\underline{T_{REF}}$

Study	Joint	UMNS	Perturbation	Loop	Task	EMG	Parameters
Patterson 2022 [74]	Elbow	CVA (C)	Stretch (S+F)	OL	Relax	Par. Task	$\underline{T_K}$, $\underline{T_{REF}}$, $\underline{EMG-P}$
Pennati 2016 [75]	Wrist	CVA (SA)	Stretch (S+F)	OL	Relax	-	I, $\underline{T_K}$, $\underline{T_B}$, $\underline{T_{REF}}$, $\underline{T_{INT-N}}$
Pennati 2023 [76]	Ankle	CVA (C)	RH stretch Stretch (S+F)	OL	Relax	Par.	I, $\underline{T_K}$, $\underline{T_B}$, $\underline{T_{REF}}$, $\underline{EMG-P}$
Pierce 2008-1 [77]	Knee	SCI (C)	Stretch (S+M+F)	OL	Relax	Par. Task	$\underline{T_{TOT}}$, $\underline{EMG-P}$
Pierce 2008-2 [78]	Knee	CP	Stretch (S+M+F)	OL	Relax	Par. Task	$\underline{T_{TOT}}$, $\underline{EMG-P}$
Pierce 2010 [79]	Knee	CP	Stretch (S+F)	OL	Relax	Par. Task	$\underline{T_{TOT}}$, $\underline{EMG-P}$
Plantin 2019 [80]	Wrist	CVA (SA)	Stretch (S+F)	OL	Relax	-	I, $\underline{T_K}$, $\underline{T_B}$, $\underline{T_{REF}}$
Rabita 2005 [81]	Ankle	CVA (NS) + SCI (NS) + TBI	Stretch (S+M+F)	OL	Relax	Par.	$\underline{T_K}$, $\underline{T_{REF}}$, $\underline{EMG-P}$
Salehi Dehno 2020 [82]	Wrist	CVA (SA)	Stretch (S+M+F)	OL	Relax	-	T_K, $\underline{T_{REF}}$
Sangani 2009 [83]	Elbow & Shoulder	CVA (NS)	Stretch (S+M)	OL	Relax + Move	Par. Task	T_K, $\underline{T_{INT-N}+T_{REF}}$[g], $\underline{EMG-P}$
Starsky 2005 [84]	Elbow	CVA (C)	Stretch (S+M)	OL	Relax	Task	T_K, $\underline{T_{REF}}$[d], $\underline{AoC}$
Tanikawa 2022 [85]	Ankle	CVA (NS)	Stretch (S+F)	OL	Relax	-	$\underline{T_K}$, $\underline{T_{REF}}$
van der Krogt 2015 [86]	Wrist	CVA (C)	Stretch (S+F)	OL	Relax + EMG	Task Mod.	$\underline{K}$, $\underline{AoC}$, $\underline{\Delta t}$
van de Ruit 2023 [87]	Elbow	CVA (C) + CP	Stretch (S+F)	OL	Relax	-	$\underline{K}$, $\underline{T_{REF}}$
van der Velden 2022-1 [88]	Elbow	CVA (C)	Stretch (S+F)	OL	Relax	-	$\underline{K}$, $\underline{T_{REF}}$
van der Velden 2022-2 [89]	Elbow	CVA (C) + CP	Stretch (S+F)	OL	Relax	-	$\underline{K}$, $\underline{T_{REF}}$
Wang 2017 [90]	Wrist	CVA (C)	Stretch (S+F)	OL	Relax	-	I, $\underline{T_K}$, $\underline{T_B}$, $\underline{T_{REF}}$
Wang 2018 [91]	Wrist	CVA (C)	Stretch (S+F)	OL	Relax	-	I, $\underline{T_K}$, $\underline{T_B}$, $\underline{T_{REF}}$
Wu 2005 [92]	Knee & Hip & Ankle	SCI (C)	Stretch (S+M+F)	OL	Relax	Par.	T_K, $\underline{T_{REF}}$, $\underline{EMG-P}$
Wu and Schmidt 2010 [93]	Hip & Knee	SCI(C)	Stretch (M)	OL	Relax	Par. Task	T_K, $\underline{T_{REF}}$, $\underline{EMG-P}$

Study	Joint	UMNS	Perturbation	Loop	Task	EMG	Parameters
Xu 2020 [94]	Ankle	CP	Stretch (S+M+F)	OL	Relax	Task	$\underline{K}$, $\underline{B}$, $\underline{T_{REF}}$
**System Modeling**							
Andringa 2021 [42]	Wrist	CVA (C)	Stretch (S+F)	OL	Relax	Mod.	I, K, $\underline{T_K}$, MAD, $\underline{T_{REF}}$
de Gooijer-van de Groep 2013 [95]	Ankle	CP	Stretch (S+M+F)	OL	Relax	Mod. Task	I, $\underline{K}$, $\underline{B}$, MAD, $\underline{T_{REF}}$
de Gooijer-van de Groep 2016 [96]	Wrist	CVA (C)	Stretch (S+F)	OL	Relax	Mod.	I, $\underline{K}$, $\underline{MAD}$, $\underline{T_{REF}}$
de Gooijer-van de Groep 2018 [97]	Wrist	CVA (SA)	Stretch (S+F)	OL	Relax	Mod.	I, $\underline{K}$, MAD, $\underline{T_{REF}}$
de Vlugt 2010 [98]	Ankle	CVA (C)	Stretch (S+M+F)	OL	Relax	Mod. Task	$\underline{I}$, T_{INT-N}, $\underline{K}$, $\underline{B}$, $\underline{MAD}$, $\underline{T_{REF}}$
Kamper 2006 [62]	MCP joint	CVA (C)	PRBS (2°)	OL	Relax	Mod.	I, $\underline{T_{INT-N}}$, $\underline{K}$, $\underline{B}$, k_p, k_v
Shorter 2021 [99]	Ankle	CVA (C)	Stretch (F, 2°)	OL	Move	Par.	$\underline{I}$, $\underline{K}$, $\underline{B}$, $\underline{EMG-T}$
Sloot 2015 [100]	Ankle (& Knee)	CP	Stretch (S+F)	OL	Relax	Mod.	$\underline{K}$, MAD, $\underline{T_{INT-N}}$, $\underline{T_{REF}}$
Wang 2017 [90]	Wrist	CVA (C)	Stretch (S+F)	OL	Relax	-	I, $\underline{T_K}$, $\underline{K}$, $\underline{T_B}$, $\underline{B}$, $\underline{k_p}$, $\underline{k_v}$, $\underline{MAD}$, Δt $\underline{T_{REF}}$
Wang 2018 [91]	Wrist	CVA (C)	Stretch (S+F)	OL	Relax	-	I, $\underline{T_K}$, $\underline{K}$, $\underline{T_B}$, $\underline{B}$, $\underline{k_p}$, $\underline{k_v}$, $\underline{MAD}$, Δt $\underline{T_{REF}}$
Zhang 2013 [101]	Ankle	CVA (C) + TBI (C)	WN (12Hz, 3°)	OL	Relax + Torque	-	I, $\underline{T_{INT-N}}$, $\underline{K}$, $\underline{B}$, $\underline{k_p}$, $\underline{k_v}$, Δt
Zhang 2014 [102]	Knee	MS	WN(10Hz, 5deg)	OL	Relax + Torque	-	I, $\underline{T_{INT-N}}$, $\underline{K}$, $\underline{B}$, $\underline{k_p}$, $\underline{k_v}$, Δt
**System Identification - IRF**							
Alibiglou 2008 [103]	Ankle + Elbow	CVA (C)	PRBS (1.7°)	OL	Relax	Task	I, $\underline{K}$, B, $\underline{k_v}$, Δt, MAD
Galiana 2005 [104]	Ankle	CVA (NS)	PRBS (1.7°)	OL	Relax	Par.	I, $\underline{K}$, $\underline{B}$, k_v, Δt, $\underline{T_K}$, $\underline{T_B}$, $\underline{T_{REF}}$, $\underline{EMG-P}$, $\underline{EMG-T}$, MAD
Mirbagheri 2007 [105]	Elbow	CVA (C)	PRBS (1.7°)	OL	Relax	Task	I, $\underline{K}$, B, $\underline{k_v}$, Δt, MAD
Mirbagheri 2008 [106]	Ankle	CVA (C)	PRBS (1.7°)	OL	Relax	Task	I, $\underline{K}$, $\underline{B}$, $\underline{k_v}$, Δt, MAD

Study	Joint	UMNS	Perturbation	Loop	Task	EMG	Parameters
Mirbagheri 2009 [107]	Elbow + Ankle	CVA (NS)	PRBS (1.7°)	OL	Relax	Task	I, $\underline{K}$, B, $\underline{k_v}$, Δt, MAD
Mirbagheri 2010 [108]	Ankle	SCI (C)	PRBS (1.7°)	OL	Relax	Task	I, $\underline{K}$, B, $\underline{k_v}$, Δt, MAD
Mirbagheri 2015 [109]	Ankle	SCI (C)	PRBS (1.7°)	OL	Relax	Task	I, $\underline{K}$, B, $\underline{k_v}$, Δt, MAD
**System Identification - FRF**							
Burne 2005 [110]	Elbow	CVA (C)	WN (3Hz, 15°)	OL	Relax + EMG	Task Mod.	$\underline{EMG-T}$, $\underline{K}$, $\underline{k_p}$
Meskers 2009 [111]	Wrist	CVA (NS)	Torque MS (3.1 - 50Hz)	CL	Position	Mod.	I, $\underline{K}$, $\underline{B}$, $\underline{k_v}$, Δt, MAD
Selves 2023 [112]	Wrist	CVA (NS)	Sine waves (3-12Hz, 10°)	OL	Relax	-	I, $\underline{K}$, $\underline{B}$
Stikvoort-Garcia 2024 [113]	Wrist	ALS	Torque MS (40Hz)	CL	Relax + Torque + Position	Mod.	I, $\underline{K}$, $\underline{B}$, $\underline{k_v}$, $\underline{k_p}$, $\underline{k_f}$, Δt, MAD, $\underline{T_{REF}}$
Van der Krogt 2015 [86]	Wrist	CVA (C)	Torque MS (50Hz)	CL	Position	-	I, K, B, $\underline{k_v}$, MAD, Δt
Van de Ruit 2023 [87]	Elbow (& Shoulder)	CVA (C) + CP	Torque MS (12Hz)	CL	Relax + Position	-	I, $\underline{K}$, $\underline{B}$

[a] CVA = Cerebrovascular Incident, SCI = Spinal Cord Injury, TBI = Traumatic Brain Injury, MS = Multiple Sclerosis, CP = Cerebral Palsy, ALS = Amyotrophic Lateral Sclerosis

[b] Use of EMG-data refers to using EMG data during controlled joint perturbation trial. If a separate research line contains neurophysiological assessment (e.g. H-reflex) this is not considered as use of EMG-data for neuromechanics quantification in this review.

[c] Only parameters obtained during controlled perturbation experiments were listed.

[d] Torques defined per unit of rotation ($\frac{Nm}{deg}$ or $\frac{Nm}{rad}$) are defined as measures of torque unless explicitly mentioned to model an intrinsic stiffness (K) or phasic reflex gain (k_p).

[e] In the case of cyclic stretches rather than a constant velocity ramp-and-hold, an average velocity was calculated based on the amplitude and frequency of the stretch-cycle.

[f] Centen et al. report max. rotation angle and max. velocity reached, in a constant torque stretch. These are interpreted as analogous to T_K and T_{REF} respectively.

[g] T_{INT-N} and T_{REF} are assessed as a combined contribution but interpreted separately based on their phasing with respect to joint rotation.

Supplementary Material 3 - Overview of reliability assessments

Table 2: Reliability scores per study and parameter. Studies are only included in this table if an explicit reliability test is performed.

**Study**	**Parameter**[a]	**Reliability scores**[b]	
		ICC	MDC
Andringa 2019	T_K	0.95	70%
	T_B	0.84	139%
	T_{REF}	0.93	89%
Centen 2017	T_K	0.86	
	T_{REF}	0.90	
Gäverth 2013	T_K	0.88	80%
	T_B	0.90	90%
	T_{REF}	0.96	40%
Mizuno 2016	T_K	0.96	
	T_{REF}	0.97	
Pennati 2023	T_K	0.90	
	T_B	0.61	
	T_{REF}	0.90	
Salehi Dehno 2020	T_{REF}	0.85	30%
van der Krogt 2015	K	0.81	86%
	AoC	0.67	78%
v.d. Velden 2022-2	K	0.97	100%
	T_{REF}	0.95	64%
de Gooijer-van de Groep 2013	K	0.93	
	T_{REF}	0.80	
de Gooijer-van de Groep 2016	K		130%
	T_{REF}		174%
Sloot 2015	K	0.99	9.4%
	T_{REF}	0.83	89%
	T_{INT-N}	0.77	80%
Selves 2023	K	0.50	29%
	B	0.94	20%
Van der Krogt 2015	k_v	0.52	947%
Van de Ruit 2023[a]	K	0.79	87%
	B	0.59	145%

[a] If multiple definitions or quantification methods for a single parameter are used, the highest reliability scores are extracted.
[b] If multiple forms of ICC are reported, the 'same day test-retest' is extracted.
[c] All MDC values are normalized to the mean or median value of that parameter reported in the study.

Supplementary Material 4 - Overview of validity assessments

Table 3: Validity matrix per study and parameter. Studies are only listed if an explicit validity test is performed. Underlined parameters indicate a test outcome aligned with hypotheses regarding UMNS pathology, i.e. a 'affirmative' validity test. If multiple quantifications for a single parameter are used in a study, the most beneficial validity outcome is extracted. PvC = A comparison between paretic and control participants or limbs. CS = is a test for correspondence with a clinical scale measuring the same construct. EMG = correspondence to EMG data, used to validate kinetic parameters reflecting neural contributions. TR = 'Treatment Response', a test for showing expected effects to UMNS treatment.

Study	PvC[a]	CS[b]	EMG	TR
**System Quantification**				
Andringa 2019	$\underline{T_K}$, TB, $\underline{T_{REF}}$	$\underline{T_K}$, $\underline{T_B}$, $\underline{T_{REF}}$		
Andringa 2020	$\underline{T_K}$, TB, $\underline{T_{REF}}$			
Andringa 2021		$\underline{T_K}$, $\underline{T_{REF}}$		
Andringa 2022		TK, TB, $Tref$		
Annaswamy 2007		$\underline{T_{TOT}}$, $\underline{AoC}$		
Bhadane 2015		$\underline{T_{REF}}$		
Boi 2024		$\underline{T_{REF}}$, $\underline{EMG-P}$	$Tref$	
Centen 2017	$\underline{T_K}$, $\underline{T_{REF}}$			
Damiano 2006	$\underline{T_{TOT}}$		T_{TOT}	
Dehno 2019	TK, $\underline{T_{REF}}$			
Gäverth 2014				$\underline{T_{REF}}$
Grey 2008	$\underline{T_{TOT}}$, $EMG-P$			
Grippo 2011	$\underline{T_{TOT}}$	$\underline{T_{TOT}}$		
Hameau 2014				$\underline{T_{REF}}$, $\underline{AoC}$
Hoffmann 2009		$\underline{T_{REF}}$		
Hornby 2006			$\underline{T_{REF}}$	
Hyngstrom 2010	$\underline{T_{REF}}$, $\underline{T_{INT-N}}$, $\underline{EMG-P}$			
Kachmar 2016				$\underline{T_{REF}}$
Kachmar 2018				$\underline{T_{REF}}$
Kamper 2006	$\underline{T_{REF}}$, Δt			
Kim 2005	$\underline{T_{TOT}}$, $\underline{EMG-P}$, $\underline{AoC}$	$\underline{T_{TOT}}$, $\underline{AoC}$		
Leng 2019	$\underline{T_K}$, $\underline{T_B}$, $\underline{T_{REF}}$	TK, TB, $Tref$		
Lindberg 2011	TK, TB, $\underline{T_{REF}}$	TK, TB, $\underline{T_{REF}}$	$\underline{T_{REF}}$	
Lorentzen 2010	TK, $\underline{T_{REF}}$, $\underline{EMG-P}$	$\underline{T_{REF}}$		
Mizuno 2016	$\underline{T_K}$, $\underline{T_{REF}}$			
Nam 2017		$\underline{T_{TOT}}$, $\underline{AoC}$		
Onushko 2010	$\underline{T_{REF}}$, $\underline{EMG-P}$, $\underline{T_{INT-N}}$			
Palmcrantz 2018				$\underline{T_{REF}}$
Pennati 2016	$\underline{T_K}$, $\underline{T_B}$, $\underline{T_{REF}}$	$Tref$		
Pennati 2023	TK, TB, $\underline{T_{REF}}$	TK, $\underline{T_{REF}}$	$\underline{T_{REF}}$	

Study	PvC	CS	EMG	TR
Pierce 2008-1	T_{TOT}, $EMG-P$	T_{TOT}	T_{TOT}	
Pierce 2008-2	$\underline{T_{TOT}}$, $\underline{EMG-P}$	$\underline{T_{TOT}}$, $EMG-P$		
Pierce 2010			T_{TOT}	
Plantin 2019		$\underline{T_{REF}}$		
Rabita 2005	TK, $\underline{T_{REF}}$	$\underline{T_{REF}}$	$\underline{T_{REF}}$	
Sangani 2009	$\underline{T_{REF}}$, $\underline{T_{INT-N}}$, $\underline{EMG-P}$			
Starsky 2005		$\underline{T_{REF}}$, $\underline{AoC}$		
Tanikawa 2022	TK, $\underline{T_{REF}}$	TK, $\underline{T_{REF}}$		
van der Krogt 2015	$\underline{K}$, $\underline{AoC}$	$\underline{K}$, AoC		
v.d. Velden 2022-2	$\underline{K}$, $\underline{T_{REF}}$	$\underline{T_{REF}}$		
Xu 2020	$\underline{K}$, $\underline{B}$, $\underline{T_{REF}}$			
**System Modeling**				
Andringa 2021		TK, $\underline{T_{REF}}$		
de Gooijer-van de Groep 2013	$\underline{K}$, B, $\underline{T_{REF}}$	K, B, $Tref$		
de Gooijer-van de Groep 2016	$\underline{K}$, $\underline{T_{REF}}$	$\underline{K}$, $\underline{T_{REF}}$		
de Vlugt 2010	$\underline{K}$, $\underline{B}$, $\underline{T_{REF}}$	$\underline{K}$, $\underline{T_{REF}}$		
Kamper 2006	$\underline{K}$, B, $\underline{T_{INT-N}}$	$\underline{K}$, B, $\underline{T_{INT-N}}$		
Shorter 2021	$\underline{K}$, B	K, B		
Sloot 2015	$\underline{K}$, $\underline{T_{REF}}$, $\underline{T_{INT-N}}$	K, $Tref$, $\underline{T_{INT-N}}$		
Wang 2017	$\underline{K}$, B, $\underline{MAD}$			
Wang 2018				$\underline{T_{REF}}$
Zhang 2013	$\underline{K}$, $\underline{B}$, k_p, $\underline{k_v}$			
Zhang 2014	K, B, k_p, $\underline{k_v}$			
**System Identification - IRF**				
Alibiglou 2008	$\underline{K}$, $\underline{k_v}$	K, k_v		
Galiana 2005	K, B, $\underline{k_v}$			
Mirbagheri 2007	$\underline{K}$, B, $\underline{k_v}$			
Mirbagheri 2008	$\underline{K}$, B, $\underline{k_v}$			
Mirbagheri 2009	$\underline{K}$, B, $\underline{k_v}$			
Mirbagheri 2010				$\underline{k_v}$
Mirbagheri 2015				$\underline{K}$, $\underline{k_v}$
**System Identification - FRF**				
Burne 2005	$\underline{K}$, $\underline{k_p}$, $\underline{EMG-T}$			
Meskers 2009	$\underline{K}$, $\underline{k_v}$			
Selves 2023	$\underline{K}$, $\underline{B}$	$\underline{K}$, B		
Stikvoort-Garcia 2024	K, B, k_v, k_p, $\underline{k_f}$, $\underline{T_{REF}}$	K, B, k_v, k_p, $\underline{k_f}$, $\underline{T_{REF}}$		
Van der Krogt 2015	k_v	k_v		
Van de Ruit 2023	$\underline{K}$, $\underline{B}$			

[a] If multiple 'Non-Paretic' controls are used (i.e. both the non-paretic limb of patients and fully healthy controls), the comparison to healthy controls was listed.

[b] If a comparison to multiple clinical tests is performed, the comparison to the clinical test most on the 'Body Function and Structures' level, was listed.
